# Human miRNA Precursors with Box H/ACA snoRNA Features

**DOI:** 10.1371/journal.pcbi.1000507

**Published:** 2009-09-18

**Authors:** Michelle S. Scott, Fabio Avolio, Motoharu Ono, Angus I. Lamond, Geoffrey J. Barton

**Affiliations:** 1 Division of Biological Chemistry and Drug Discovery, College of Life Sciences, University of Dundee, Dundee, United Kingdom; 2 Wellcome Trust Centre for Gene Regulation and Expression, College of Life Sciences, University of Dundee, Dundee, United Kingdom; Bar-Ilan University, Israel

## Abstract

MicroRNAs (miRNAs) and small nucleolar RNAs (snoRNAs) are two classes of small non-coding regulatory RNAs, which have been much investigated in recent years. While their respective functions in the cell are distinct, they share interesting genomic similarities, and recent sequencing projects have identified processed forms of snoRNAs that resemble miRNAs. Here, we investigate a possible evolutionary relationship between miRNAs and box H/ACA snoRNAs. A comparison of the genomic locations of reported miRNAs and snoRNAs reveals an overlap of specific members of these classes. To test the hypothesis that some miRNAs might have evolved from snoRNA encoding genomic regions, reported miRNA-encoding regions were scanned for the presence of box H/ACA snoRNA features. Twenty miRNA precursors show significant similarity to H/ACA snoRNAs as predicted by snoGPS. These include molecules predicted to target known ribosomal RNA pseudouridylation sites *in vivo* for which no guide snoRNA has yet been reported. The predicted folded structures of these twenty H/ACA snoRNA-like miRNA precursors reveal molecules which resemble the structures of known box H/ACA snoRNAs. The genomic regions surrounding these predicted snoRNA-like miRNAs are often similar to regions around snoRNA retroposons, including the presence of transposable elements, target site duplications and poly (A) tails. We further show that the precursors of five H/ACA snoRNA-like miRNAs (miR-151, miR-605, mir-664, miR-215 and miR-140) bind to dyskerin, a specific protein component of functional box H/ACA small nucleolar ribonucleoprotein complexes suggesting that these molecules have retained some H/ACA snoRNA functionality. The detection of small RNA molecules that share features of miRNAs and snoRNAs suggest that these classes of RNA may have an evolutionary relationship.

## Author Summary

The major functions known for RNA were long believed to be either messenger RNAs, which function as intermediates between genes and proteins, or ribosomal RNAs and transfer RNAs which carry out the translation process. In recent years, however, newly discovered classes of small RNAs have been shown to play important cellular roles. These include microRNAs (miRNAs), which can regulate the production of specific proteins, and small nucleolar RNAs (snoRNAs), which recognise and chemically modify specific sequences in ribosomal RNA. Although miRNAs and snoRNAs are currently believed to be generated by different cellular pathways and to function in different cellular compartments, members of these two types of small RNAs display numerous genomic similarities, and a small number of snoRNAs have been shown to encode miRNAs in several organisms. Here we systematically investigate a possible evolutionary relationship between snoRNAs and miRNAs. Using computational analysis, we identify twenty genomic regions encoding miRNAs with highly significant similarity to snoRNAs, both on the level of their surrounding genomic context as well as their predicted folded structure. A subset of these miRNAs display functional snoRNA characteristics, strengthening the possibility that these miRNA molecules might have evolved from snoRNAs.

## Introduction

Small nucleolar RNAs (snoRNAs) and microRNAs (miRNAs) are two classes of abundant non-coding regulatory RNAs that carry out fundamental cellular activities but that have only been comprehensively investigated in recent years. SnoRNAs are small RNA molecules of approximately 60–300 nucleotides in length which generally serve as guides for the catalytic modification of selected ribosomal RNA nucleotides [1],[2]. SnoRNAs associate with specific proteins, which are conserved amongst all eukaryotes, to form small nucleolar ribonucleoparticles (snoRNPs). Two main groups of snoRNAs have been described. The box C/D snoRNAs, which bind the four conserved core box C/D snoRNP proteins fibrillarin, NOP56, NOP5/NOP58 and NHP2L1, are involved in 2′-O-ribose methylation. The box H/ACA snoRNAs, which bind the four conserved core box H/ACA snoRNP proteins DKC1 (dyskerin), GAR1, NHP2 and NOP10, catalyse pseudouridylation. In vertebrates, most snoRNAs have been shown to reside in introns of protein coding host genes and are processed out of the excised introns [3]. However, two box C/D snoRNAs have recently been found to be transcribed from independent RNA pol II units [4].

MiRNAs are ∼18–24 nucleotide-long RNAs that are processed out of ∼70 nucleotide-long hairpin structures (called pre-miRNAs) [5]. In mammals, miRNAs have been shown to be involved mainly in mRNA translation inhibition [6] although recently, they have also been reported to activate translation [7]. A large class of miRNAs are encoded in introns of protein-coding genes and are co-expressed with these host genes [8]–[10]. The remaining miRNAs are encoded in independent transcription units. Some of these miRNAs have been shown to be under the control of the RNA polymerase II [11] while others are transcribed by the RNA polymerase III [12].

Many members of the snoRNA and miRNA classes are well conserved throughout evolution [1],[2],[13]. Correspondence between several yeast and human snoRNAs and their target sites have been established and many snoRNAs have a very high sequence identity within mammals as shown in the snoRNAbase database [14]. In the case of miRNAs, several families have been found to be well conserved in metazoans [13],[15]. However, recent reports also suggest the existence of species- and lineage-specific snoRNAs and miRNAs [13],[16],[17]. These and other reports on their origin and evolution are providing clues about the emergence of large groups of these recently evolved molecules. Through bioinformatic searches, Weber [17] and Luo and Li [16] identified hundreds of human snoRNAs and snoRNA-related molecules that are derived from transposable elements (TEs), thus confirming the widespread nature of this phenomenon, initially described for a small number of snoRNAs [2],[18]. These analyses suggest that many snoRNAs result from the retroposition of existing snoRNAs that used long interspersed nuclear element (LINE) machinery to transpose themselves to new genomic locations. Many of these snoRNA-related molecules are surrounded by the presence of sequence features typical of retrogenes such as target site duplications (TSDs) and poly (A) tails at their 3′ end. These snoRNA retroposition events generated hundreds of sno-related molecules, termed snoRTs (snoRNA retroposons) by Weber [17], many of which had never been previously identified, but some of which were previously described as functional snoRNAs [16]. SnoRNA retroposition thus not only permits maintenance of a pool of intact snoRNA copies to safeguard against the effects of deleterious mutations but could possibly also allow for the creation of regulatory RNA molecules that might bind new targets [17]. Given the stringent thresholds used to search for snoRNA copies in both studies, it is likely that many more such molecules exist in the human genome but might have diverged further from their parental copies and are yet to be discovered.

Recent reports have also described some miRNAs as being derived from TEs, suggesting a possible mechanism for the rapid generation of miRNAs and their corresponding target sites. In the first such report, Smalheiser and Torvik identified six miRNAs that are derived from TEs [19]. Two subsequent studies identified a further 95 [12] and 55 [20],[21] known miRNAs that might be derived from TEs as well as an additional 85 predicted novel TE-derived miRNA genes [20]. The TEs that are most frequently found in association with miRNAs are the L2 and MIR families [20]. As TEs are the most non-conserved sequence elements in eukaryotic genomes [22], the generation of miRNAs through TEs represents a mechanism that could be a driving force in speciation events and evolution by rapidly creating new regulatory elements in the control of protein production [19],[20].

A recent report investigating the small RNAs present in human cells has demonstrated the existence of specific small RNA fragments derived from larger known non-coding RNA molecules [23]. In particular, distinct small fragments of sizes between 23 and 25 nucleotides were found to map to four box H/ACA snoRNAs [23] (listed in Table 1). In addition to this, Ender and colleagues have recently reported eight box H/ACA snoRNA-derived miRNA-like molecules that can be immunoprecipitated with Ago proteins [24]. While these short H/ACA snoRNA-derived fragments might be discounted merely as non-functional degradation products, several unrelated observations suggest otherwise. Firstly, only specific fragments derived from one region of each snoRNA were identified, rather than a ladder of fragments consistent with degradation. Secondly, other snoRNAs encode smaller fragments that are stably produced. Indeed, three miRNAs present in the miRNA repository miRBase [25] can be shown to be encoded in known H/ACA snoRNAs (listed in Table 1). Although at least one pair of these miRNAs and snoRNAs are known to be co-localised in the genome as mentioned in miRBase [25], it is not known whether the processing of these molecules is independent or dependent and sequential. Thirdly, as mentioned above, miRNA and snoRNA members have both been found to be TE-derived, suggesting a similar origin and evolution for at least some members of these small non-coding RNA classes. Here, in light of the accumulation of data suggesting a connection between box H/ACA snoRNAs and miRNA-like molecules, we investigate the possibility of an evolutionary relationship between members of these classes of RNA.

**Table 1:** Small fragments generated from snoRNAs.

Box H/ACA snoRNA	Chromosome	Genomic Coordinates of snoRNA	Other H/ACA snoRNAs with same predicted rRNA target site	Known miRNA encoded within snoRNA	Genomic Coordinates of miRNA hairpin	Smaller fragment detected	Length of small fragment
ACA36B	1	218440511–218440641	ACA36, ACA8, ACA50, ACA62, SNORA36C	mir-664	218440503–218440584	mir-664 [24],[58]	23
HBI-61	3	187987158–187987335	-	mir-1248	187987155–187987260	mir-1248 [58]	27
ACA34	12	47334432–47334568	ACA2A, ACA2B	mir-1291	47334494–47334580	mir-1291 [58]	24
ACA7	3	12856811–12856949	ACA7B	-	N/A	[23]	25
ACA7B	3	130598743–130598881	ACA7	-	N/A	[23]	25
U17b	1	28707657–28707861	unknown target	-	N/A	[23]	23
U71a	20	36489363–36489500	U71b, U71c, U71d	-	N/A	[23]	24
ACA45	15	81221751–81221877	-	ACA45 sRNA	N/A	ACA45 sRNA [24]	23
ACA47	17	72596984–72597170	-	-	N/A	ACA47 sRNA [24]	22
HBI-100	1	174204156–174204299	ACA12	-	N/A	HBI-100 sRNA [24]	22
ACA56	X	153656467–153656595	-	-	N/A	ACA56 sRNA [24]	23
ACA3	11	8662350–8662479	-	-	N/A	ACA3 sRNA [24]	23
ACA50	16	57151201–57151336	ACA62, ACA8, ACA36, ACA36B	-	N/A	ACA50 sRNA [24]	24
U92	9	19053654–19053784	-	-	N/A	U92 sRNA [24]	24

## Results

### Smaller RNA species are encoded in box H/ACA snoRNAs

A comparison between the genomic positions of reported miRNA genes from miRbase [25] and box H/ACA snoRNAs reveals three occurrences of overlap between these RNA species (Table 1, top section). In all three cases, between 75% (mir-1291) and 97% (mir-1248) of the miRNA hairpin is contained within the snoRNA (using the coordinates of the UCSC Genome Browser as described in the Methods). Moreover, in all three cases, greater than 90% of the mature miRNA as defined in miRbase release 11.0 [25] is contained within the snoRNA.

In addition to these known miRNAs encoded in box H/ACA snoRNAs, ten small fragments matching exactly to portions of eleven box H/ACA snoRNAs have been detected [23],[24] and are listed in Table 1 (bottom section). One of the fragments is identical to two very similar H/ACA snoRNAs, ACA7 and ACA7B. Of these ten small fragments, seven have been shown to be bound by Ago proteins and one of these, ACA45 sRNA, has experimentally validated targets [24].

Apart from HBI-100, all box H/ACA snoRNAs from Table 1 that contain experimentally detected smaller fragments are either experimentally verified snoRNAs or close paralogues of such experimentally validated snoRNAs. ACA34, ACA45, ACA47, ACA56, ACA3, ACA50, ACA7, HBI-61, U17b, U71a and U92 have been shown experimentally to display characteristics of H/ACA snoRNAs [26]–[31]. ACA36B is a close paralogue (88% identity) of the experimentally validated H/ACA snoRNA ACA36 [28]. ACA7B is a close paralogue of ACA7 (98% identical). ACA7B and ACA36B share both their predicted rRNA targets with ACA7 and ACA36 respectively, as described in the snoRNAbase [14]. In addition, as shown in Table 1, seven of the box H/ACA snoRNAs that encode smaller experimentally detected fragments share their predicted rRNA and snRNA targets with other box H/ACA snoRNAs and one (U17b) does not have a known target. The remaining six box H/ACA snoRNAs, HBI-61, ACA45, ACA47, ACA56, ACA3 and U92, are predicted to guide the pseudouridylation of known modified residues [14] and no other snoRNA is known to serve as a guide for these residues.

**Figure 1:**
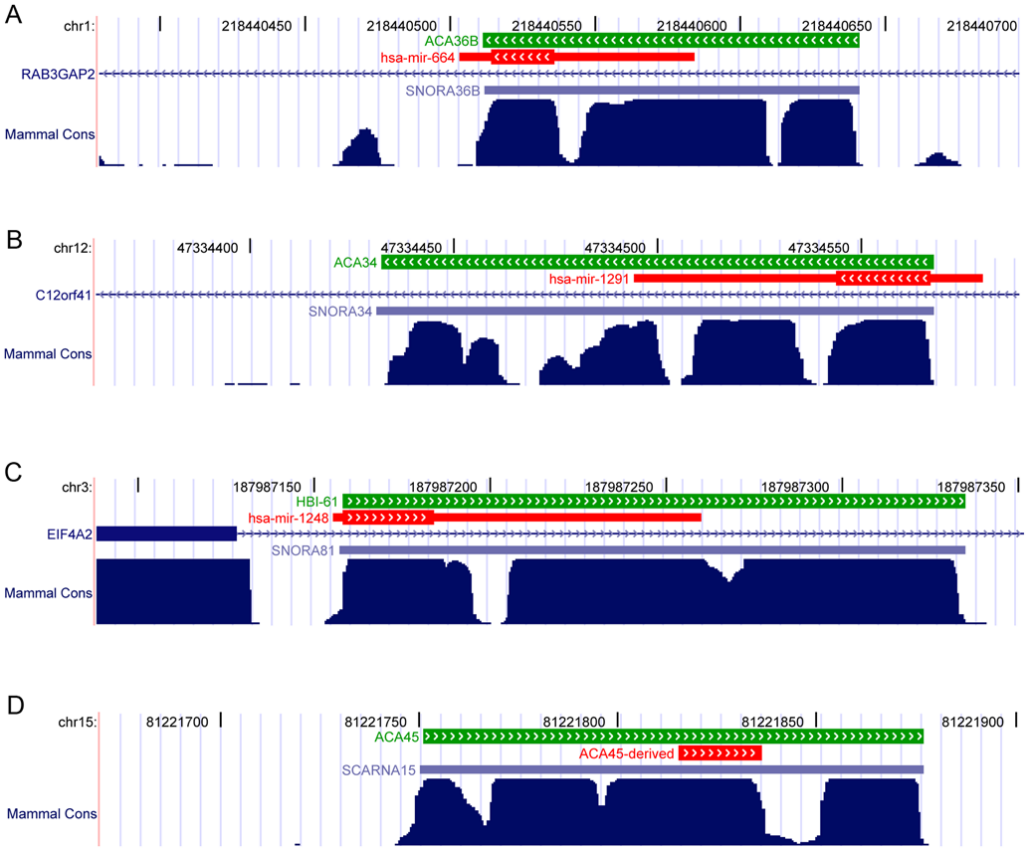
Conservation of the box H/ACA snoRNAs that encode miRNAs. Screenshots of the UCSC Genome Browser [48] displaying RefSeq genes (blue lines with hatch marks), miRNA hairpins (red blocks with hatch marks indicating the mature portion) and snoRNAs (green blocks with hatch marks) are displayed above the mammalian conservation track [50] for the genomic regions surrounding ACA36B (A), ACA34 (B), HBI-61 (C) and ACA45 (D).

The UCSC Genome Browser mammalian conservation track shows that for all snoRNAs listed in Table 1 except U71a and ACA56, the conserved region around these molecules covers the entire snoRNA molecules, not only the miRNA hairpins or small RNA fragments detected (Figure 1 and Figure S1). The miRNA hairpins of miR-664 and miR-1291 have short 3′ and 5′ regions respectively that do not overlap with a snoRNA. These regions correspond to the least well conserved regions of the whole miRNA/snoRNA molecules. This suggests that these regions originally encoded snoRNAs and not necessarily miRNAs in the most recent common ancestor. Indeed, to our knowledge, apart from mir-664 and ACA45 sRNA, none of the other miRNAs and smaller fragments have been detected in other mammalian species, suggesting the capability of generating smaller RNA molecules from these snoRNAs might be a recent event.

### miRNAs are encoded in genomic regions predicted as H/ACA snoRNAs

The box H/ACA snoRNA/miRNA relationship described above was further investigated by studying all known miRNAs to determine whether they might be encoded within genomic regions predicted to harbour H/ACA snoRNAs. Indeed, if some miRNAs have evolved from H/ACA snoRNA encoding regions, they might still display snoRNA features. The mammalian version of the snoGPS program predicts pseudouridylation guides in human, mouse and rat genomes by scoring weakly conserved primary and secondary structure motifs using a deterministic search algorithm [32]. The mammalian version of the snoGPS program also includes a cross-species implementation (snoGPS-C) which takes account of conservation between several mammalian genomes to predict box H/ACA snoRNAs [32]. A locally-installed copy of the mammalian snoGPS program was used to scan with the two-hairpin model for the presence of box H/ACA snoRNAs in 676 distinct sequences consisting of human miRNAs from miRBase (version 11.0) [25] and an additional 175 padding nucleotides upstream and downstream (referred to as the extended miRNA molecules). We did not use the cross-species implementation of snoGPS (snoGPS-C) because many of the newly described snoRNAs (especially the TE-derived snoRNAs) are lineage- or species-specific [17].

**Figure 2:**
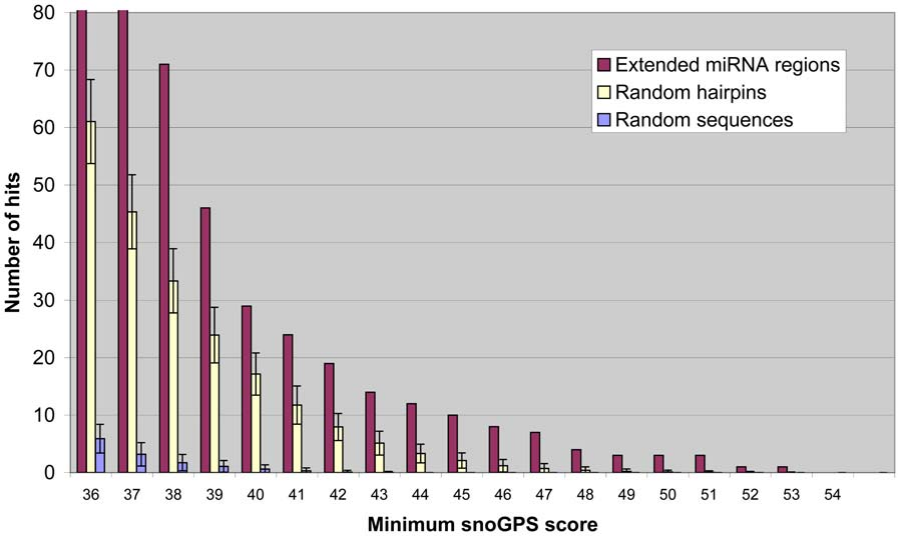
Number of snoGPS hits above given scores. The number of snoGPS hits above scores ranging from 36 to 54 is shown for 676 extended miRNA regions (red), sets of randomly-generated sequences (blue) and sets of randomly-generated hairpins (yellow). For the randomly-generated sequences and hairpins, 100 sets of 676 molecules were run and the average values are shown here. The error bars represent standard deviation.

In order to investigate whether the number of snoGPS predicted hits above a certain threshold was significant, we used snoGPS to scan 100 sets of 676 randomly generated sequences of same length distribution as the miRNAs under study, as described in the Methods section. The number of hits above a given threshold for both the set of extended miRNA sequences under study and the randomly generated sequences is shown in Figure 2. MiRNA precursors, like H/ACA snoRNAs, consist of at least one hairpin. To control for this, a second set of control sequences consisting of 676 randomly generated hairpins of same length distribution and minimum free-energy distribution (as calculated by RNAfold [33]) as the miRNA hairpins under study was generated. 100 such random hairpin sets were scanned using snoGPS and their average number of hits is shown in Figure 2. As expected, the number of hits for the random hairpin groups is significantly higher than the number of hits for the random sequence groups that have not been constrained to form hairpins. However, for all hit score thresholds investigated, the number of hits predicted by snoGPS for the miRNA group is significantly higher than the number of hits predicted for any of the randomly generated groups. This suggests that genomic regions around a significant number of miRNAs contain features that very closely resemble box H/ACA snoRNAs.

148 distinct extended miRNA molecules were predicted to encode at least one hit above a score of 35.0, which is the threshold that is ‘typically’ used when predicting new candidate snoRNAs by snoGPS-C [32]. Since we chose to use the original snoGPS version, we set the threshold higher, at 40.0, in order to consider only very likely candidates. Taking this conservative threshold, 29 distinct extended miRNA molecules were predicted to encode at least one hit. All predicted hits were folded to reveal their predicted secondary structure, using RNAstructure [34]. When the highest snoGPS hit could not be folded in a secondary structure that was within 10% of the lowest RNAstructure predicted minimum free-energy, snoGPS hits of lower score (but still above 40) were considered. The best snoGPS hit is defined as the snoGPS hit with highest score that has a predicted secondary structure minimum free-energy within 10% of the lowest predicted minimum free-energy structure for this molecule. Twenty extended miRNA regions had best snoGPS hits above a score of 40.0 and the remaining nine extended miRNA regions with lower best snoGPS hits were not considered further. Table 2 describes the best hit for each of these twenty extended miRNA molecules.

**Table 2:** Best snoGPS hits.

miRNA	Overlaping known H/ACA snoRNA	Highest snoGPS hit	Best snoGPS hit^a^	Number of mature sequence reads [reference]^b^	Number of star sequence reads [reference]	Predicted pseudouridylation site of best hit	Other snoRNAs that target same site^c^
mir-1291	ACA34	53.1	53.1	6 [58]	N/A	LSU.U4259	ACA34
mir-664	ACA36B	51.43	51.43	193 [58]	N/A	SSU.U1244	ACA36B, ACA36, SNORA36C
mir-548d-1	N/A	51.2	51.2	5 [46]	2 [46]	LSU.U3608	ACA19
mir-151	N/A	48.22	43.81	>5000 [58]	∼750 [58]	5SU.U55	U72
mir-548d-2	N/A	47.4	47.4	4 [46]	2 [46]	LSU.U4596	HBI-61
mir-215	N/A	47.35	47.35	85 [58]	N/A	LSU.3699	ACA19
mir-549	N/A	46.44	46.44	1 [45]	N/A	SSU.U1696	**unknown guide**
mir-1258	N/A	45.93	45.69	15 [58]	N/A	LSU.U1723	ACA52
mir-885	N/A	45.86	45.86	260 [58]	2 [58]	LSU.U1656	ACA56
mir-140	N/A	44.15	44.15	>40000 [58]	10 [58]	LSU.U4491	**unknown guide**
mir-1248	HBI-61	43.76	43.76	6 [58]	N/A	LSU.U4596	HBI-61
mir-1255b-2	N/A	42.77	42.77	9 [58]	N/A	LSU.U3699	ACA19
mir-548f-5	N/A	41.83	40.06	5 [58]	N/A	LSU.U4596	HBI-61
mir-1262	N/A	41.69	41.69	52 [58]	N/A	U3.U12	**unknown guide**
mir-1266	N/A	41.37	41.37	208 [58]	N/A	LSU.U4927	ACA17
mir-1297	N/A	41.06	41.06	10 [58]	N/A	LSU.U1723	ACA52
mir-548m	N/A	40.93	40.93	2 [58]	N/A	LSU.U4536	ACA40
mir-605	N/A	40.53	40.53	1 [45]	N/A	LSU.U4491	**unknown guide**
mir-520a	N/A	40.37	40.37	>1000 [58]	∼40 [58]	LSU.3787	ACA48, HBI-43
mir-616	N/A	40.11	40.11	5 [58]	N/A	LSU.U3813	ACA58

a highest snoGPS hit with predicted folded structure within 10% of lowest RNAstructure predicted free-energy structure.

b count includes isomiRs [58] when available.

c according to snoRNAbase [14].

The position of the predicted H/ACA snoRNA was compared to the position of the miRNA hairpin, taking coordinates downloaded from the UCSC Table Browser as described in the Methods. Apart from the predicted snoRNAs that contain mir-151 and mir-215, all predicted snoRNAs in Table 2 contain at least 90% of their encoded miRNA hairpins. Approximately 80% of the hairpins of both mir-151 and mir-215 are contained in their respective predicted snoRNA. In addition, for all miRNAs described in Table 2, at least 90% of the mature miRNA is contained within the predicted snoRNA. In this respect, all these snoRNA-predicted miRNA pairs are similar to the known H/ACA snoRNAs that encode smaller fragments detected experimentally, described in Table 1.

SnoGPS predicts guide sequences and corresponding rRNA pseudouridylation sites within snoRNAs. For all the best snoGPS hits with scores above 40 listed in Table 2, the predicted pseudouridylation sites are reported. While most of these predicted pseudouridylation sites are known to be recognised by already reported box H/ACA snoRNAs, four are labelled as having an unknown guide in snoRNAbase [14]. Indeed, the H/ACA snoRNAs predicted in the extended region around mir-549, mir-140, mir-1262 and mir-605 are all predicted to serve as guides for experimentally validated pseudouridylation sites whose guides are unknown, making these interesting candidates for further studies. Some of these might represent genomic regions with a dual function, serving both to produce miRNAs and snoRNAs.

The miRNAs reported in miRbase have not all been validated to the same extent. While the mature forms of some of the miRNAs have only been identified with a very small number of sequence reads, others have been identified by larger numbers of reads, display characteristic miRNA signatures (with detection of a much smaller number of star reads than the mature form reads [24],[35]) and have been functionally validated. For each of the twenty miRNAs described in Table 2, we include the number of sequence reads and when available, the number of star reads, as reported in the literature. Ten of the miRNAs in Table 2 have been identified with at least 10 reads and four of these (miR-151, miR-885, miR-140 and miR-520a) also have corresponding star reads of lower abundance. On the other hand, three reported miRNAs with snoRNA-like features, miR-549, miR-548m and miR-605, have been identified with fewer than 4 reads.

While the best snoGPS hit has been investigated here, it is important to point out that some extended miRNA regions obtain more than one high-scoring hit. Most notably, mir-548d-1 and mir-548d-2 have high-scoring hits in both their hairpins, in a manner reminiscent of well validated H/ACA snoRNAs such as E2 and U65.

### Structures of the predicted snoRNA-like miRNA precursors

**Figure 3:**
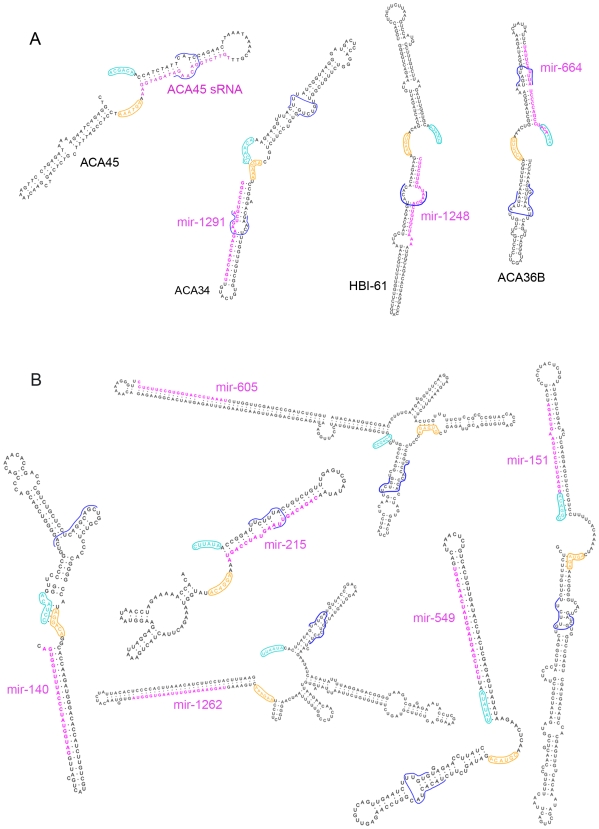
Secondary structure predictions of H/ACA snoRNAs. The secondary structure predictions of known H/ACA snoRNAs encoding experimentally detected miRNAs (A) as well as predicted H/ACA snoRNAs encoding known miRNAs (B) were drawn using RNAstructure [34] and RNAviz [51]. Mature miRNAs are drawn in pink. H and ACA boxes are shown respectively in orange and cyan. Guide regions are outlined using dark blue lines.

Box H/ACA snoRNAs have very distinct features. They usually consist of two hairpins, each of which is followed by short single-stranded regions (the H and ACA boxes). While the H box is located between the two hairpins, the ACA box is located at the 3′ end of the molecule. One or both of the hairpins contain bulges, allowing base-pairing with the target RNA, in complex pseudo-knot structures. In order to better characterise the predicted snoRNAs encoding miRNAs and visualise the position of the mature miRNA within these molecules, all predicted snoRNA sequences were folded using RNAstructure [34] and are shown in Figure 3B and Figure S2. In addition, the predicted secondary structure of the four snoRNAs encoding known miRNAs (from Table 1) are also shown (Figure 3A). Most of the predicted snoRNAs encoding miRNAs resemble typical snoRNAs with two main hairpins, characteristic boxes and one or two bulges containing the predicted RNA target complementary sites

### Repeat elements in proximity of snoRNA-like miRNAs

**Figure 4:**
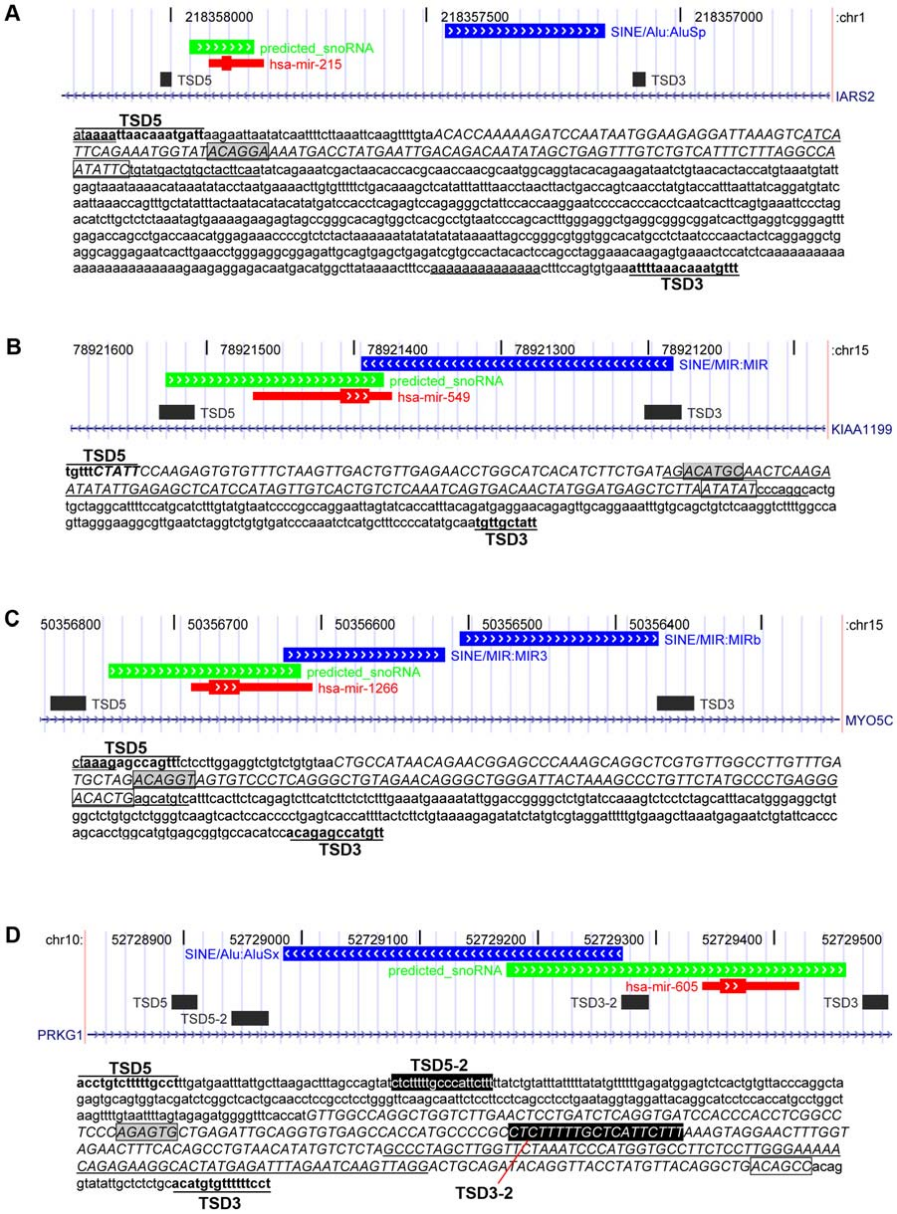
Retrogene-like structures encoding miRNAs. Screenshots of the UCSC Genome Browser [48] displaying RefSeq genes (blue lines with hatch marks), miRNA hairpins (red blocks), snoRNAs (green blocks with hatch marks), repeat-elements (blue blocks with hatch marks) and TSDs (black blocks) are shown for the genomic regions surrounding mir-215 (A), mir-549 (B), mir-1266 (C) and mir-605 (D). The thick regions in the red blocks represent the mature regions of the miRNAs. The 5′ TSD is annotated as TSD5 and the 3′ TSD is annotated as TSD3. Shown below the genomic structure illustrations are the sequences corresponding to these regions. In the sequences, the miRNA hairpins are underlined, the predicted snoRNAs are shown in uppercase italics, the boxes ACA and H are respectively shown in a box and a shaded box and putative poly(A) tails are underlined using a wavy line.

Because numerous snoRNAs and miRNAs have been described as being derived from TEs, all extended miRNA molecules predicted to have box H/ACA snoRNA features surrounding them (from Table 2) were further investigated for the presence of repeat elements using RepeatMasker (http://www.repeatmasker.org). Sixteen of the twenty miRNAs originally considered have repeat elements either overlapping the predicted snoRNA encoding the miRNA or within 400 nucleotides. The position of the repeat elements with respect to the position of the miRNA and predicted snoRNA is shown in Figure 4 and Figure S3. In addition, putative L1 consensus recognition sites and flanking target site duplications (TSDs), which are characteristic of retrogenes, were also identified surrounding many of these molecules (Figure 4 and Figure S4). Some of these putative snoRNA-encoded miRNA regions have a genomic structure that is very similar to numerous snoRTs [16], consisting of the snoRNA/miRNA region in close proximity to a downstream SINE member repeat element and flanked by target site duplications (TSDs). In addition, immediately upstream from the 5′ TSD, an L1 consensus recognition site is often found and a poly (A) tail can be identified upstream from the 3′ TSD. Three such examples resembling the HBI-61c snoRT from [16] are shown in Figure 4A–C. Shown in Figure 4D is the genomic region surrounding mir-605, which consists of two pairs of TSDs, one of which flanks a SINE repeat element and the other of which flanks the whole snoRNA/miRNA/SINE region. This structure resembles the ACA18e snoRT example from [16]. The examples shown in Figure 4 suggest that it is the predicted snoRNA and not the miRNA hairpin that was captured in the retrogene construct and initially transposed, in a manner similar to snoRT events previously described [16],[17].

### Predicted snoRNA-like miRNA precursors that bind dyskerin and localise in the nucleolus

**Figure 5:**
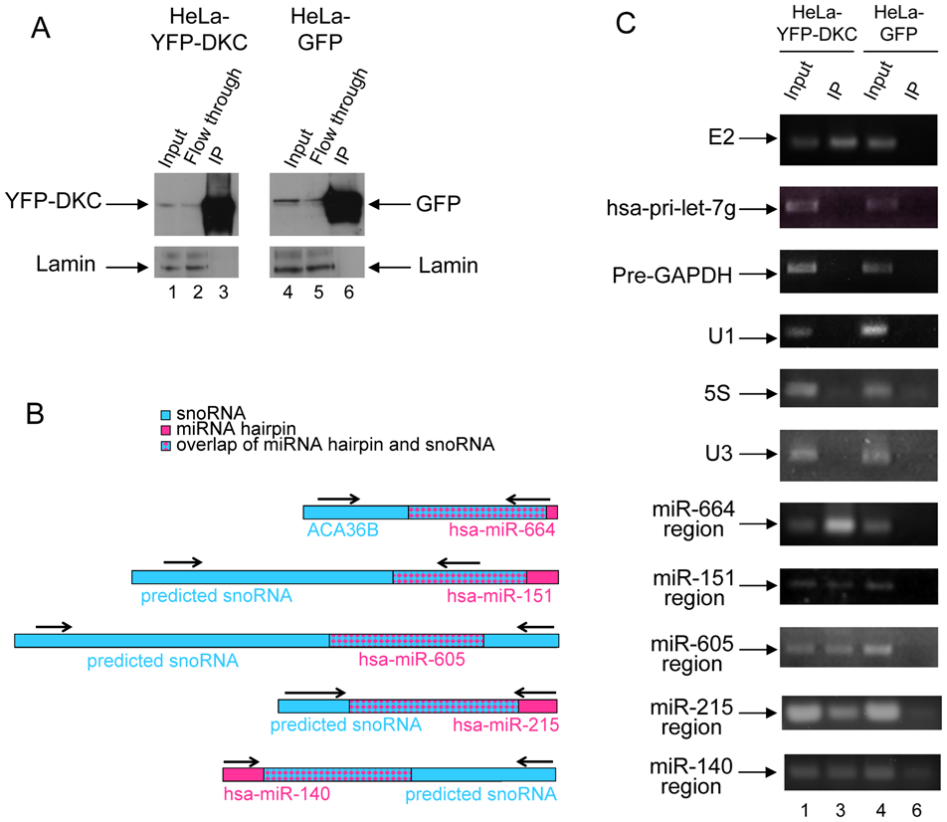
snoRNA-like miRNA precursors that bind dyskerin. Nuclear extracts were prepared from HeLa cells stably expressing either free GFP or YFP-dyskerin (YFP-DKC) and immunoprecipitated using an anti-GFP antibody. A Western blot confirming specificity of the immunoprecipitation using an anti GFP antibody. The same membrane was reprobed with an antibody against lamin as a loading control. B Position of the primers used to detect the specified miRNA extended regions. C RT-PCR used to detect co-precipitated hsa-mir-664, hsa-mir-151, hsa-mir-605, has-mir-215 and has-mir-140 miRNA precursors, with E2 box H/ACA snoRNA as positive control and hsa-pri-let-7g miRNA, U3 box C/D snoRNA, U1 snRNA, 5S rRNA and GAPDH pre-mRNA as negative controls for dyskerin-associated RNAs. The lane numbering in panel C refers to the lanes shown in panel A.

To explore further potential snoRNA-like features of these miRNA precursors and to investigate whether they have retained some H/ACA snoRNA functionality, we tested whether they can bind dyskerin, a protein component of the functional box H/ACA snoRNPs. Dyskerin serves as the pseudouridine synthase [36] and is proposed to bind the ACA box [37]. Five of the twenty snoRNA-like miRNAs, mir-664, mir-151, mir-605, mir-215 and mir-140 were selected for this analysis because they are expressed in HeLa cells. Purified nuclei from HeLa cells expressing YFP-dyskerin or GFP as a control were immunoprecipitated using an antibody against the fluorescent proteins as described in the Methods. The RNA isolated from these samples was analysed by RT-PCR for the presence of the molecules of interest. As shown in Figure 5C, in addition to E2 (a well-characterised box H/ACA snoRNA), five snoRNA-like miRNA precursors (the extended regions of mir-664, mir-151, mir-605, mir-215 and mir-140) are bound by dyskerin. In contrast, the precursor of hsa-let-7g, a miRNA precursor with no similarity to box H/ACA snoRNAs is not pulled down by dyskerin. And as expected, other abundant nuclear RNAs including GAPDH pre-mRNA, the small nuclear RNA U1, the box C/D snoRNA U3 and 5S rRNA are not immunoprecipitated by dyskerin. These binding experiments confirm the *in silico* predictions that some miRNA precursors sufficiently resemble box H/ACA snoRNAs to be bound by dyskerin.

**Figure 6:**
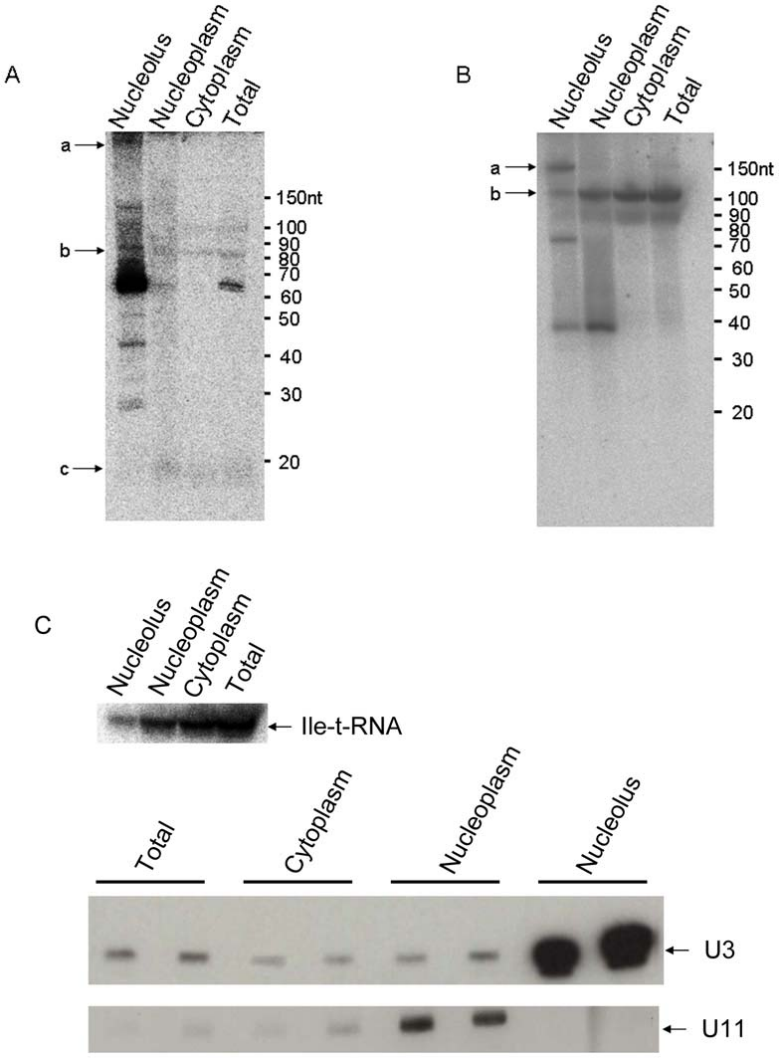
Subcellular localization of H/ACA snoRNA-like miRNA precursors. Northern blots of HeLa cell extracts fractionated into cytoplasmic, nucleoplasmic and nucleolar fractions were probed for the presence of mir-151 (A) and mir-664 (B) encoding molecules using probes against the respective mature miRNA region. In both panels A and B, bands labeled with ‘a’ represent the expected size of the predicted snoRNAs, those labeled with ‘b’ represent the expected size for the miRNA hairpins and ‘c’ represents the expected size of the mature miRNA. C As controls of the fractionation, northern blots of the same RNA preparations were probed against isoleucine tRNA, U3 and U11.

Two of the dyskerin-bound miRNA precursors were further characterised by fractionated northern analysis to investigate where the predicted snoRNAs and smaller fragments localise in the cell. As shown in Figure 6, bands of the size of the predicted H/ACA snoRNA full-length molecules encoding mir-151 and mir-664 localise to the nucleolus (bands labelled with ‘a’ in panels 6A and 6B). Bands of the size of the miRNA hairpins are detected in all three fractions although the putative mir-151 hairpin is mainly nucleolar whereas the putative mir-664 hairpin accumulates predominantly in the nucleoplasm and cytoplasm (bands labelled with ‘b’ in panels 6A and 6B). The mir-151 mature form is also detected and mainly found in the nucleoplasm and cytoplasm (bands labelled with ‘c’ in panel 6A. For a longer exposure, please see Figure S5). And a band slightly larger than the mir-664 mature form localises mainly in the nucleoplasm.

## Discussion

Numerous miRNAs have been previously shown to be repeat-derived [12], [19]–[21] and many snoRNAs have been described as retrogenes [16],[17]. Here, we hypothesize that some reported miRNAs have evolved from box H/ACA snoRNAs or snoRTs. Several lines of evidence support this possibility. Fourteen known box H/ACA snoRNAs encode smaller fragments of miRNA size that have been experimentally detected, three of which are reported miRNAs. Analysis of mammalian conservation patterns suggests that these genomic regions originally encoded the full-length H/ACA snoRNA molecules and not only the miRNAs. If a subgroup of miRNAs has indeed evolved from box H/ACA snoRNAs, we reasoned that although some of these miRNAs might have sufficiently evolved to no longer bear measurable similarity to H/ACA snoRNAs, others might display detectable H/ACA snoRNA features. In an effort to further characterise the prevalence of the relationship between these two classes of small RNA molecules, we scanned the regions encoding known miRNAs for the presence of box H/ACA snoRNA features using the snoGPS predictor. We identified twenty reported miRNAs from miRBase [25] that are encoded in larger regions predicted with high scores to be box H/ACA snoRNAs. The predicted box H/ACA snoRNAs display usual box H/ACA snoRNA features and resemble the fourteen box H/ACA snoRNAs that encode experimentally detected smaller fragments. In addition, the genomic sequence surrounding several of the predicted snoRNA-like miRNAs very closely resembles those described for some snoRTs [16]. These analyses show that some genomic regions previously reported to encode miRNAs resemble regions that encode H/ACA snoRNAs on numerous levels. This suggests that these miRNAs have evolved from H/ACA snoRNAs or snoRTs. We applied stringent selection criteria in our analysis, so anticipate that other box H/ACA snoRNA-like miRNA precursors also exist but have not been identified here.

Due to the inherent similarity between miRNAs and snoRNAs, such a relationship is easy to overlook as once a region is categorized as belonging to one molecular class, it is often no longer considered when searching for other types of molecules. The human genome has been scanned previously for the presence of box H/ACA snoRNAs using mammalian snoGPS [32]. However, the search space was limited to the 20% most well conserved regions between the human, mouse and rat genomes. In addition, the dataset was repeat-masked, thus eliminating repeat-derived regions such as those encoding many miRNAs. Finally, the dataset was restricted to sequences that do not overlap with known features in the UCSC Human Genome Browser database, thus probably eliminating all known miRNAs. As a consequence, it is not surprising that no miRNA encoding regions were identified as also encoding predicted snoRNAs. Moreover, at least one recent snoRNA predictor, SnoReport [38] uses miRNAs as negative training examples, thus making it very unlikely to identify any of the snoRNA-like miRNA regions described here.

Although no significant sequence similarity is detected between predicted snoRNA molecules encoding miRNAs and the known snoRNAs that target the same pseudouridylation sites, it is interesting to note that three snoRNAs (ACA52, HBI-61 and ACA19) share their target pseudouridylation sites with eight of the predicted snoRNAs encoding miRNAs (Table 2). This situation also exists amongst known snoRNAs, some of which share the same target site without displaying significant sequence similarity. In particular, examples exist of a snoRNA harbouring two guide regions, each of which is shared with a different snoRNA. For example, ACA22 which shares one of its targets with ACA33 and the other with U64, has no significant sequence similarity with either molecule. Similarly, ACA50 shares target sites with ACA36, ACA8 and ACA62 but while it has high sequence identity with ACA62, it has no significant sequence similarity with ACA8 or ACA36.

This redundancy in rRNA complementarity may suggest some box H/ACA snoRNAs and snoRTs are not under as much selective pressure to avoid mutations. We hypothesize that some of these snoRNA encoding regions might be in the process of evolving from functional snoRNAs to miRNA-like precursors. This process might be facilitated by the fact that box H/ACA snoRNAs have a structure (2 hairpins) that is probably favourable to the formation of miRNAs. The *in silico* data presented here support these ideas. We tested the predictions by experimentally showing that the precursors of five of the predicted snoRNA-like miRNAs, mir-664, mir-151, mir-605, mir-215 and mir-140, interact with dyskerin, a protein component of functional H/ACA snoRNPs. While a lack of interaction to dyskerin could not rule out an evolutionary relationship between these miRNAs and snoRNAs as the miRNAs might have evolved sufficiently to no longer interact with functional protein components of snoRNPs, the detection of such an interaction considerably reinforces such claims. These results show that the snoRNA-like miRNA precursors sufficiently resemble box H/ACA snoRNAs to bind dyskerin, strengthening the possibility of an evolutionary relationship between these molecules. Further experiments will be necessary to investigate whether these molecules also retain the capability of targeting rRNA *in vivo*. It will also be necessary to experimentally test whether the remaining fifteen predicted snoRNA-like miRNA precursors also display aspects of H/ACA snoRNA functionality. The fact that three of these molecules (mir-548d-1, mir-1297 and mir-616) display the sequence AGA instead of the canonical ACA box could indicate that they have evolved sufficiently to no longer retain H/ACA snoRNA functionality. We do note that while identifying molecules that display both miRNA and snoRNA functionality supports our evolutionary hypothesis, we also expect to find a larger number of molecules that display features of both molecules but do not represent completely prototypical examples.

It is interesting to note that *Saccharomyces cerevisiae* has snoRNAs but no reported miRNAs, consistent with a relationship where primordial snoRNAs may have given rise to certain classes of miRNAs. This idea is supported by a recent article by Saraiya and Wang reporting that the primitive parasitic protozoan *Giardia lamblia*, which does not have RNA interference capabilities but has miRNA processing machinery, uses box C/D snoRNAs as miRNA precursors [39]. In addition to this, Taft and colleagues have recently reported that most snoRNAs in animals, *Arabidopsis* and *Schizosaccharomyces pombe* generate small RNAs (of ∼20–24 nucleotides in length for animal box H/ACA snoRNAs), which are associated with argonaute proteins [40]. Current data such as these dual function molecules with both miRNA and snoRNA capabilities which exist in both human [24] and *Giardia* [39] and likely many other organisms [40] suggest this process of evolving from a snoRNA encoding genomic region to a miRNA-like encoding region could be ongoing.

In addition to investigating whether some of the H/ACA snoRNA-like miRNA precursors display functional H/ACA snoRNA capability by binding to dyskerin (Figure 5), we have also characterised the cellular localisation of two of these molecules: the precursors of mir-151 and mir-664 (Figure 6). While bands of the size of the predicted full-length H/ACA snoRNA molecules localise to the nucleolus, consistent with their binding to dyskerin, bands of the size of the predicted hairpin form of these miRNAs can be found in all three fractions considered but accumulate mainly in the nucleolus (in the case of mir-151) and in the nucleoplasm and cytoplasm (in the case of mir-664). The mature form of mir-151 accumulates mainly in the nucleoplasm which is unusual for a miRNA but might be a consequence of the snoRNA features displayed by its precursor. These results are consistent with a recent study showing that the precursors and/or mature form of a number of rat miRNAs accumulate in the nucleolus [41]. Further studies will be required to investigate the exact nature and role of each of these molecules in these cellular compartments as well as how they are processed.

While all the miRNAs characterised in Table 2 are classified as miRNAs in miRBase [25], they have not all been extensively analysed. Of the five extended miRNA regions that we experimentally found to be bound by dyskerin, three (mir-151, mir-140 and mir-215) have been further characterized and functionally validated, either by studies of their processing into their mature form or validation of their targets and effects. Indeed, mir-151 has been shown to be processed into its mature form by usual miRNA processing machinery [42] while functional targets of mir-140 have been experimentally validated [43]. And mir-215, which has been shown to have reduced expression in cancer tissues compared to normal cells, is capable of inducing cell-cycle arrest, colony suppression and cell detachment from a solid support when transfected into cells [44]. Mir-664 and mir-605 have not, to our knowledge, been further functionally validated and will require additional experimental evidence to confirm they are true miRNAs. In particular, mir-605 has only been identified previously with one sequence read [45]. Given that the extended region of mir-605 is predicted to serve as a guide for an experimentally validated pseudouridylation site whose guide is unknown, we postulate that this region encodes an H/ACA snoRNA rather than a miRNA. This type of analysis can thus be used to filter out unlikely miRNA candidates from the large miRNA repositories which contain many poorly characterized molecules.

A recent large-scale study defining an expression atlas for mammalian miRNAs, by Landgraf and colleagues, classifies known miRNAs into four different groups: prototypical, repeat-derived, repeat-clustered and unclassified [46]. A lack of repetitiveness, evolutionary conservation and 5′ end processing were considered to classify miRNAs as prototypical. Only two (mir-215 and mir-140) of our twenty miRNAs encoded in predicted snoRNAs are classified as protypical in this study. The remaining eighteen miRNAs were either classified as repeat-derived (5 miRNAs), repeat-clustered (1 miRNA), unclassified (2 miRNAs) or were not considered in the study (10 miRNAs). The results of a recent deep-sequencing study [45] analysed in the context of the Landgraf study reveals that only 17% of all miRNAs identified in this manner are prototypical [46]. The remaining miRNAs displayed irregularities in their processing or unusual sequence conservation patterns [46]. The authors further went on to investigate possible functional implications, determining that unlike non-prototypical miRNAs, prototypical miRNAs showed enrichment of putative target sites in 3′UTRs [46]. In light of the relationship uncovered here between box H/ACA snoRNAs and miRNAs, it is reasonable to speculate that snoRNA-encoded miRNAs might be in the process of evolving into functional miRNAs but still retain some non-miRNA-like features which could preclude them from being classified as prototypical. Alternatively, perhaps these molecules should be classified as a separate small RNA class. Further analysis will be necessary to clarify the role and exact relationship of these molecules.

## Methods

### Sequence analysis of genomic regions surrounding miRNA genes

The genomic positions, sequences and flanking regions of human miRNA genes and box H/ACA snoRNA genes were downloaded from the UCSC Table Browser [47], wgRNA table [14],[25] using the March 2006 assembly of the human genome. Repeat elements surrounding these genomic regions were identified with the RepeatMasker program (http://www.repeatmasker.org). MiRNA and box H/ACA snoRNA genes as well as repeat-elements and sequence features were visualised with the UCSC Genome Browser [48], using information from the wgRNA table [14],[25], the Vertebrate Multiz Alignment and PhastCons Conservation utilities [49],[50] as well as custom tracks.

### Prediction of H/ACA snoRNAs

676 distinct human miRNA encoding regions (including the miRNA hairpins and 175 flanking nucleotides on either side) were scanned for the presence of box H/ACA snoRNAs using a locally-installed copy of the snoGPS program [32]. In addition to known miRNAs, two sets of control sequences were also scanned using snoGPS. The first sets of control sequences consisted of 676 randomly generated sequences having the same nucleotide composition as human intronic sequences and the same length distribution as the miRNA encoding regions. The second sets of control sequences consisted of randomly generated hairpins having the same length distribution and minimum free-energy distribution as the miRNA hairpin set. RNAfold [33] was used to predict the minimum free-energy of the randomly-generated hairpin sequences. The snoGPS parameters used throughout this analysis were as follow. The target sites for pseudouridylation were defined in the file ‘snAndRrna.targ’ provided with the snoGPS download. The descriptor and scoretable files used were respectively ‘MamGUs2.v3.desc’ and ‘human.v3.scoretables’, provided with the snoGPS download.

### Secondary structure visualisation

RNA secondary structures were predicted using RNAstructure 4.5 [34] and annotated using RnaViz 2.0 [51].

### Immunoprecipitation and quantitative RT-PCR

Immunoprecipitations were prepared as previously described [52]. Nuclear lysates were prepared from HeLa^YFP-Dyskerin^ and HeLa^GFP^ stable cell lines. Purified nuclei were resuspended in RIPA buffer to solubilise proteins. Fluorescence proteins were immunoprecipitated using GFP binder (ChromoTek) covalently coupled to NHS-activated Sepharose 4 Fast Flow beads (GE Healthcare at 1 mg/ml as previously described [53]). Samples were divided in two and for Input samples RNA was isolated from one half of each nuclear lysate. RNA was isolated by the TRIzol method with DNase I treatment, according to manufacturer's instructions (Invitrogen). RT-PCR was performed to detect immunoprecipitated RNAs. Reverse transcription and PCR were performed with the following gene-specific primers (hsa-pri-let-7g: 5′-CGCTCCGTTTCCTTTTGCCTG-3′ and 5′ TACAGTTATCTCCTGTACCGG-3′, U3: 5′-AGAGGTAGCGTTTTCTCCTGAGCG-3′ and 5′ ACCACTCAGACCGCGTTCTC-3′, pre-GAPDH: 5′-CGCATCTTCTTTTGCGTCGCCAG-3′ and 5′-GGTCAATGAAGGGGTCATTGATGGC-3′, U1: 5′-TACCTGGCAGGGGAGATACCATGATC-3′ and 5′-GCAGTCGAGTTTCCCACATTTGGGG-3′, 5S: 5′-ACGCGCCCGATCTCGTCTGAT-3′ and 5′-GCCTACAGCACCCGGTATTCCC-3′, miR-664: 5′-GTGTTAAGTTCAGTTCAGGGTAG-3′ and 5′-CATTTTGTAGGCTGGGGATAAATG-3′, miR-151: 5′-GGCTTACCCTATGCTGCTATA-3′ and 5′-GTAGGGGATGAGACATACTAGAC-3′, miR-605: 5′-CTGGTCTTGAACTCCTGATCTC-3′ and 5′- GCTGTCAGCCTGTAACATAGG-3′, miR-215: 5′-CCAAAAAGATCCAATAATGGAAGAGGATTAAAG-3′ and 5′-TTGAAGTAGCACAGTCATACAG-3′, miR-140: 5′-GTGTGTCTCTCTCTGTGTCC-3′ and 5′-GGATGTCCCAAGGGGGCCAG-3′) using the SuperScript one-step RT-PCR kit (Invitrogen). To decide linearity of cycles, we performed real time PCR using the Superscript III Platinum SYBR Green one-step qRT-PCR Kit (Invitrogen) and Rotor-Gene RG-3000 system (Corbett Research). The same amount of RNA for input and immunoprecipitated RNA (IP) was used as templates for RT-PCR reactions. Each experiment was repeated three times independently.

### Northern and high sensitivity RNA blot analysis

HeLa cell extracts were fractionated using sucrose gradients, as previously described [54]–[56]. Total HeLa cell RNA and RNA from separate cytoplasmic, nucleoplasmic and nucleolar fractions was isolated using the TRIzol method, with Dnase I treatment, according to manufacturer's instructions (Invitrogen). Equal amounts of RNA from each sample were separated by 8M Urea polyacrylamide denaturing gel electrophoresis in 1×MOPS buffer and the RNA transferred onto nylon membrane (Hybond-N; Amersham) by electro blotting. After chemical cross linking, the membrane was hybridized with ^32^P 5′ end-labelled oligoribonucleotide probes specific for the following RNA species; (mir-664: 5′- UGUAGGCUGGGGAUAAAUGAAUA-3′, mir-151: 5′-CCUCAAGGAGCUUCAGUCUAG-3′, tRNA-Ile 5′-UGGUGGCCCGUACGGGGAUCGA-3′, U11: 5′-TCTTGATGTCGATTCCGCACGCAGAGCAATCGAGTTGCCC-3′ and U3: 5′-CACTCAGACCGCGTTCTCTCCCTCTCACTCCCCAATACGG-3′). High sensitivity RNA blots were prepared as previously described [57].

## Supporting Information

Figure S1 Mammalian conservation of box H/ACA snoRNAs that encode experimentally detected smaller fragments
(1.63 MB PDF)

Figure S2 Predicted secondary structures of H/ACA snoRNA-like miRNA precursors
(0.07 MB PDF)

Figure S3 Repeat elements in proximity of H/ACA snoRNA-like miRNA genomic regions
(3.58 MB PDF)

Figure S4 Sequence elements surrounding selected miRNAs
(0.03 MB PDF)

Figure S5 Northern blot showing the subcellular localization of H/ACA snoRNA-like miRNA precursors (same as Figure 6AB but longer exposure)
(0.31 MB PDF)

## References

[1] Kiss T (2002). Small nucleolar RNAs: an abundant group of noncoding RNAs with diverse cellular functions.. Cell.

[2] Bachellerie JP, Cavaille J, Huttenhofer A (2002). The expanding snoRNA world.. Biochimie.

[3] Filipowicz W, Pogacic V (2002). Biogenesis of small nucleolar ribonucleoproteins.. Curr Opin Cell Biol.

[4] Tycowski KT, Aab A, Steitz JA (2004). Guide RNAs with 5′ caps and novel box C/D snoRNA-like domains for modification of snRNAs in metazoa.. Curr Biol.

[5] Lai EC (2005). miRNAs: whys and wherefores of miRNA-mediated regulation.. Curr Biol.

[6] Lai EC (2003). microRNAs: runts of the genome assert themselves.. Curr Biol.

[7] Vasudevan S, Tong Y, Steitz JA (2007). Switching from repression to activation: microRNAs can up-regulate translation.. Science.

[8] Baskerville S, Bartel DP (2005). Microarray profiling of microRNAs reveals frequent coexpression with neighboring miRNAs and host genes.. Rna.

[9] Kim YK, Kim VN (2007). Processing of intronic microRNAs.. Embo J.

[10] Rodriguez A, Griffiths-Jones S, Ashurst JL, Bradley A (2004). Identification of mammalian microRNA host genes and transcription units.. Genome Res.

[11] Lee Y, Kim M, Han J, Yeom KH, Lee S (2004). MicroRNA genes are transcribed by RNA polymerase II.. Embo J.

[12] Borchert GM, Lanier W, Davidson BL (2006). RNA polymerase III transcribes human microRNAs.. Nat Struct Mol Biol.

[13] Niwa R, Slack FJ (2007). The evolution of animal microRNA function.. Curr Opin Genet Dev.

[14] Lestrade L, Weber MJ (2006). snoRNA-LBME-db, a comprehensive database of human H/ACA and C/D box snoRNAs.. Nucleic Acids Res.

[15] Hertel J, Lindemeyer M, Missal K, Fried C, Tanzer A (2006). The expansion of the metazoan microRNA repertoire.. BMC Genomics.

[16] Luo Y, Li S (2007). Genome-wide analyses of retrogenes derived from the human box H/ACA snoRNAs.. Nucleic Acids Res.

[17] Weber MJ (2006). Mammalian Small Nucleolar RNAs Are Mobile Genetic Elements.. PLoS Genet.

[18] Vitali P, Royo H, Seitz H, Bachellerie JP, Huttenhofer A (2003). Identification of 13 novel human modification guide RNAs.. Nucleic Acids Res.

[19] Smalheiser NR, Torvik VI (2005). Mammalian microRNAs derived from genomic repeats.. Trends Genet.

[20] Piriyapongsa J, Marino-Ramirez L, Jordan IK (2007). Origin and evolution of human microRNAs from transposable elements.. Genetics.

[21] Piriyapongsa J, Jordan IK (2007). A family of human microRNA genes from miniature inverted-repeat transposable elements.. PLoS ONE.

[22] Lander ES, Linton LM, Birren B, Nusbaum C, Zody MC (2001). Initial sequencing and analysis of the human genome.. Nature.

[23] Kawaji H, Nakamura M, Takahashi Y, Sandelin A, Katayama S (2008). Hidden layers of human small RNAs.. BMC Genomics.

[24] Ender C, Krek A, Friedlander MR, Beitzinger M, Weinmann L (2008). A Human snoRNA with MicroRNA-Like Functions.. Mol Cell.

[25] Griffiths-Jones S, Grocock RJ, van Dongen S, Bateman A, Enright AJ (2006). miRBase: microRNA sequences, targets and gene nomenclature.. Nucleic Acids Res.

[26] Ganot P, Caizergues-Ferrer M, Kiss T (1997). The family of box ACA small nucleolar RNAs is defined by an evolutionarily conserved secondary structure and ubiquitous sequence elements essential for RNA accumulation.. Genes Dev.

[27] Gu AD, Zhou H, Yu CH, Qu LH (2005). A novel experimental approach for systematic identification of box H/ACA snoRNAs from eukaryotes.. Nucleic Acids Res.

[28] Kiss AM, Jady BE, Bertrand E, Kiss T (2004). Human box H/ACA pseudouridylation guide RNA machinery.. Mol Cell Biol.

[29] Kiss T, Filipowicz W (1993). Small nucleolar RNAs encoded by introns of the human cell cycle regulatory gene RCC1.. Embo J.

[30] Ruff EA, Rimoldi OJ, Raghu B, Eliceiri GL (1993). Three small nucleolar RNAs of unique nucleotide sequences.. Proc Natl Acad Sci U S A.

[31] Darzacq X, Jady BE, Verheggen C, Kiss AM, Bertrand E (2002). Cajal body-specific small nuclear RNAs: a novel class of 2′-O-methylation and pseudouridylation guide RNAs.. Embo J.

[32] Schattner P, Barberan-Soler S, Lowe TM (2006). A computational screen for mammalian pseudouridylation guide H/ACA RNAs.. Rna.

[33] Hofacker IL, Fontana W, Stadler PF, Bonhoeffer LS, Tacker M (1994). Fast folding and comparison of RNA secondary structures.. Monatshefte für Chemie.

[34] Mathews DH, Disney MD, Childs JL, Schroeder SJ, Zuker M (2004). Incorporating chemical modification constraints into a dynamic programming algorithm for prediction of RNA secondary structure.. Proc Natl Acad Sci U S A.

[35] Bartel DP (2004). MicroRNAs: genomics, biogenesis, mechanism, and function.. Cell.

[36] Hoang C, Ferre-D'Amare AR (2001). Cocrystal structure of a tRNA Psi55 pseudouridine synthase: nucleotide flipping by an RNA-modifying enzyme.. Cell.

[37] Li L, Ye K (2006). Crystal structure of an H/ACA box ribonucleoprotein particle.. Nature.

[38] Hertel J, Hofacker IL, Stadler PF (2008). SnoReport: computational identification of snoRNAs with unknown targets.. Bioinformatics.

[39] Saraiya AA, Wang CC (2008). snoRNA, a Novel Precursor of microRNA in Giardia lamblia.. PLoS Pathog.

[40] Taft RJ, Glazov EA, Lassmann T, Hayashizaki Y, Carninci P (2009). Small RNAs derived from snoRNAs.. Rna.

[41] Politz JC, Hogan EM, Pederson T (2009). MicroRNAs with a nucleolar location.. Rna.

[42] Kawahara Y, Zinshteyn B, Chendrimada TP, Shiekhattar R, Nishikura K (2007). RNA editing of the microRNA-151 precursor blocks cleavage by the Dicer-TRBP complex.. EMBO Rep.

[43] Nicolas FE, Pais H, Schwach F, Lindow M, Kauppinen S (2008). Experimental identification of microRNA-140 targets by silencing and overexpressing miR-140.. Rna.

[44] Braun CJ, Zhang X, Savelyeva I, Wolff S, Moll UM (2008). p53-Responsive micrornas 192 and 215 are capable of inducing cell cycle arrest.. Cancer Res.

[45] Cummins JM, He Y, Leary RJ, Pagliarini R, Diaz LA (2006). The colorectal microRNAome.. Proc Natl Acad Sci U S A.

[46] Landgraf P, Rusu M, Sheridan R, Sewer A, Iovino N (2007). A mammalian microRNA expression atlas based on small RNA library sequencing.. Cell.

[47] Karolchik D, Hinrichs AS, Furey TS, Roskin KM, Sugnet CW (2004). The UCSC Table Browser data retrieval tool.. Nucleic Acids Res.

[48] Kent WJ, Sugnet CW, Furey TS, Roskin KM, Pringle TH (2002). The human genome browser at UCSC.. Genome Res.

[49] Blanchette M, Kent WJ, Riemer C, Elnitski L, Smit AF (2004). Aligning multiple genomic sequences with the threaded blockset aligner.. Genome Res.

[50] Siepel A, Bejerano G, Pedersen JS, Hinrichs AS, Hou M (2005). Evolutionarily conserved elements in vertebrate, insect, worm, and yeast genomes.. Genome Res.

[51] De Rijk P, Wuyts J, De Wachter R (2003). RnaViz 2: an improved representation of RNA secondary structure.. Bioinformatics.

[52] Trinkle-Mulcahy L, Andersen J, Lam YW, Moorhead G, Mann M (2006). Repo-Man recruits PP1 gamma to chromatin and is essential for cell viability.. J Cell Biol.

[53] Rothbauer U, Zolghadr K, Muyldermans S, Schepers A, Cardoso MC (2008). A versatile nanotrap for biochemical and functional studies with fluorescent fusion proteins.. Mol Cell Proteomics.

[54] Andersen JS, Lam YW, Leung AK, Ong SE, Lyon CE (2005). Nucleolar proteome dynamics.. Nature.

[55] Andersen JS, Lyon CE, Fox AH, Leung AK, Lam YW (2002). Directed proteomic analysis of the human nucleolus.. Curr Biol.

[56] Lam YW, Lamond AI, Mann M, Andersen JS (2007). Analysis of nucleolar protein dynamics reveals the nuclear degradation of ribosomal proteins.. Curr Biol.

[57] Pall GS, Codony-Servat C, Byrne J, Ritchie L, Hamilton A (2007). Carbodiimide-mediated cross-linking of RNA to nylon membranes improves the detection of siRNA, miRNA and piRNA by northern blot.. Nucleic Acids Res.

[58] Morin RD, O'Connor MD, Griffith M, Kuchenbauer F, Delaney A (2008). Application of massively parallel sequencing to microRNA profiling and discovery in human embryonic stem cells.. Genome Res.

