# Supplementary material for: Human miRNA Precursors with Box H/ACA snoRNA Features
Source: PLoS Comput Biol. 2009 Sep 18;5(9):e1000507. doi: 10.1371/journal.pcbi.1000507 (PMC2730528; doi:10.1371/journal.pcbi.1000507)
Supplement: Figure S1 — Mammalian conservation of box H/ACA snoRNAs that encode experimentally detected smaller fragments (1.63 MB PDF) [file pcbi.1000507.s001.pdf]

Screenshots of the UCSC Genome Browser displaying RefSeq genes (dark blue lines with hatch marks), short snoRNA generated fragments (brown blocks with hatch marks) and snoRNAs (green blocks with hatch marks) are displayed above the mammalian conservation track for the genomic regions surrounding the specified snoRNA.

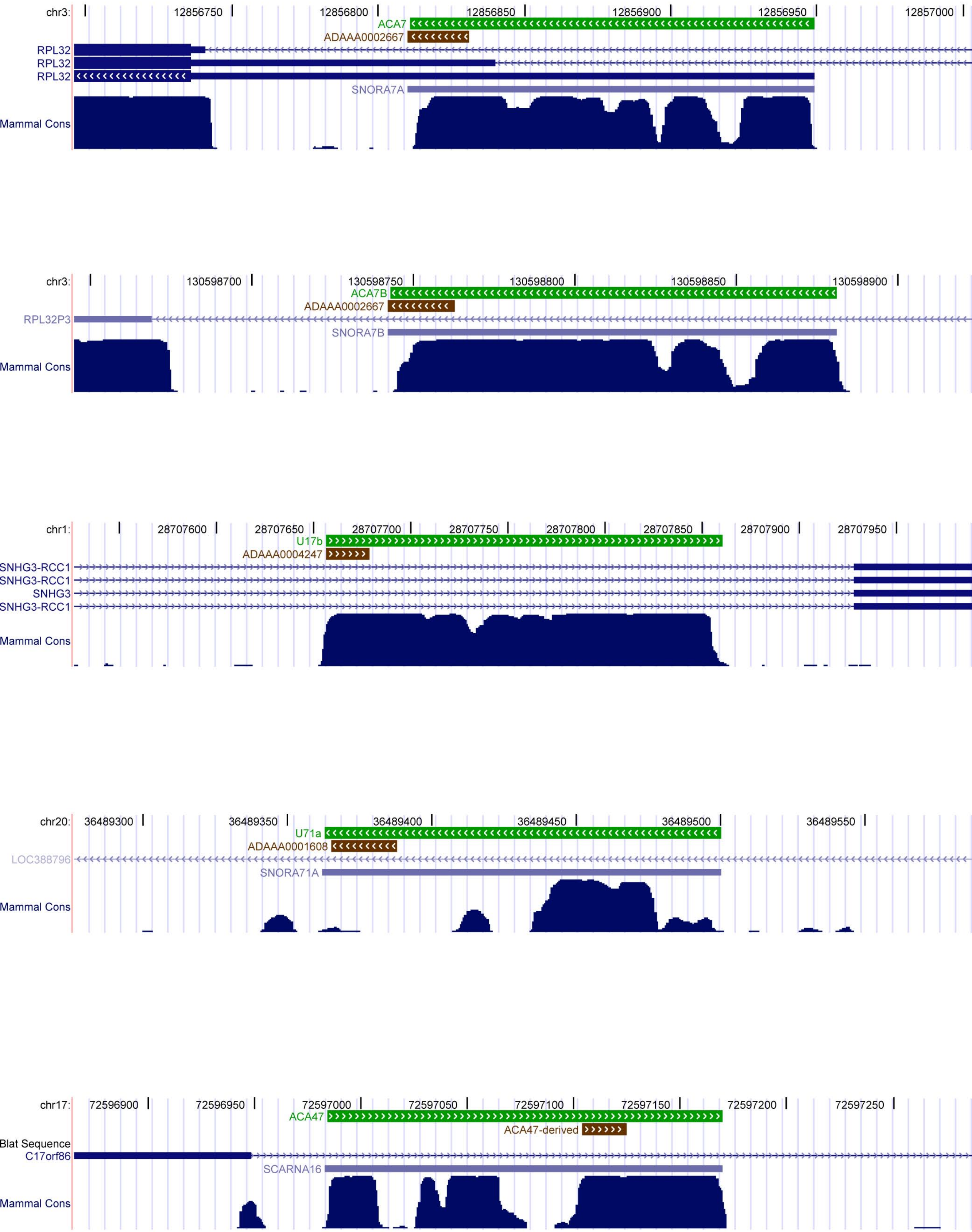

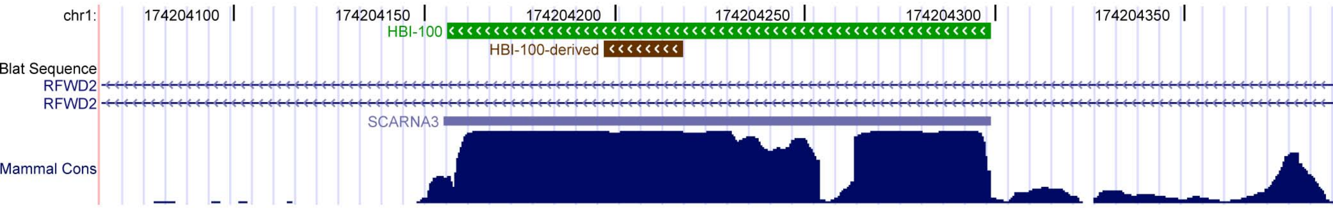
chr1:
174204100
174204150
174204200
174204250
174204300
174204350
HBI-100
HBI-100-derived
Blat Sequence
RFWD2
RFWD2
SCARNA3
Mammal Cons

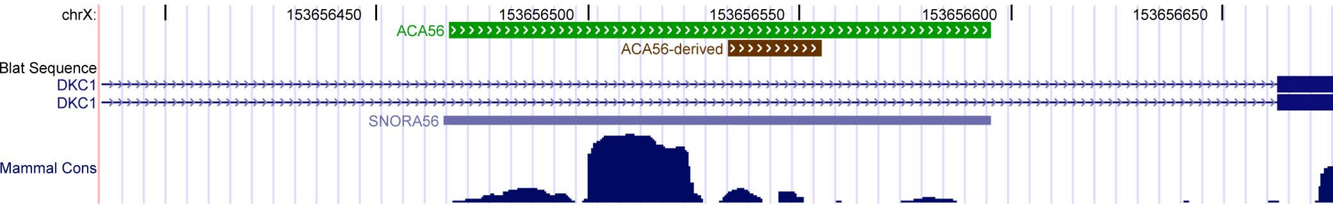
chrX:
153656450
153656500
153656550
153656600
153656650
ACA56
ACA56-derived
Blat Sequence
DKC1
DKC1
SNORA56
Mammal Cons

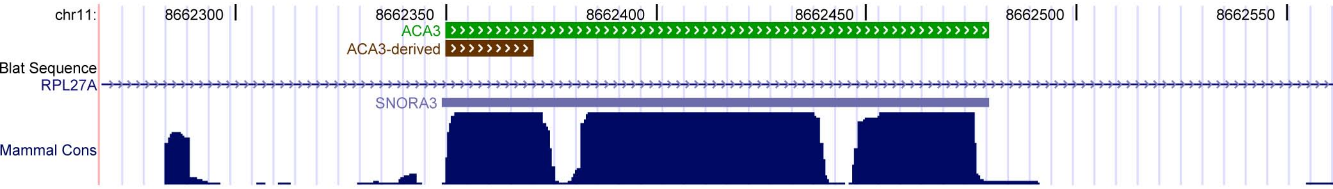
chr11:
8662300
8662350
8662400
8662450
8662500
8662550
ACA3
ACA3-derived
Blat Sequence
RPL27A
SNORA3
Mammal Cons

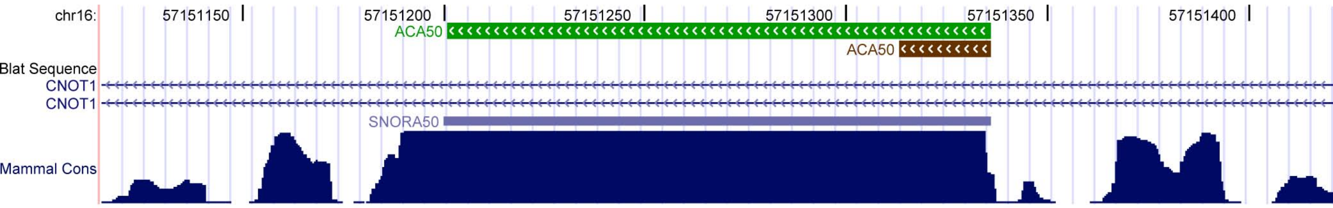
chr16:
57151150
57151200
57151250
57151300
57151350
57151400
ACA50
ACA50
Blat Sequence
CNOT1
CNOT1
SNORA50
Mammal Cons

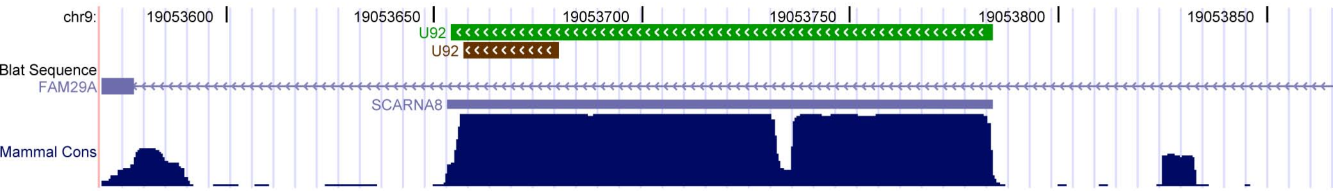
chr9:
19053600
19053650
19053700
19053750
19053800
19053850
U92
U92
Blat Sequence
FAM29A
SCARNA8
Mammal Cons
