# Supplementary figures and images for: Human miRNA Precursors with Box H/ACA snoRNA Features
Source: PLoS Comput Biol. 2009 Sep 18;5(9):e1000507. doi: 10.1371/journal.pcbi.1000507 (PMC2730528; doi:10.1371/journal.pcbi.1000507)

mir-548d-1

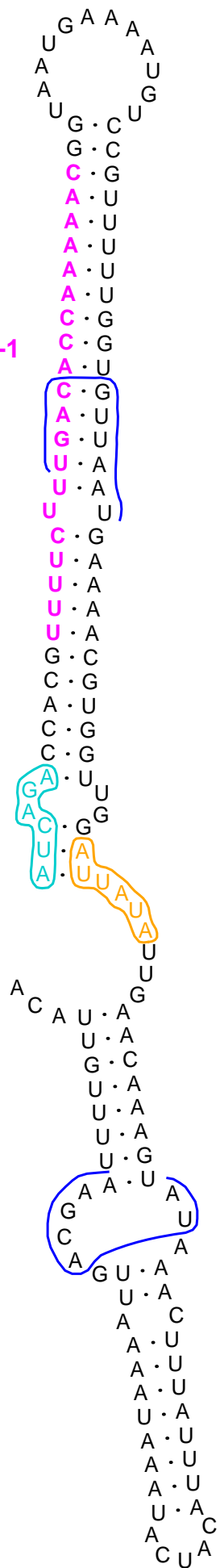

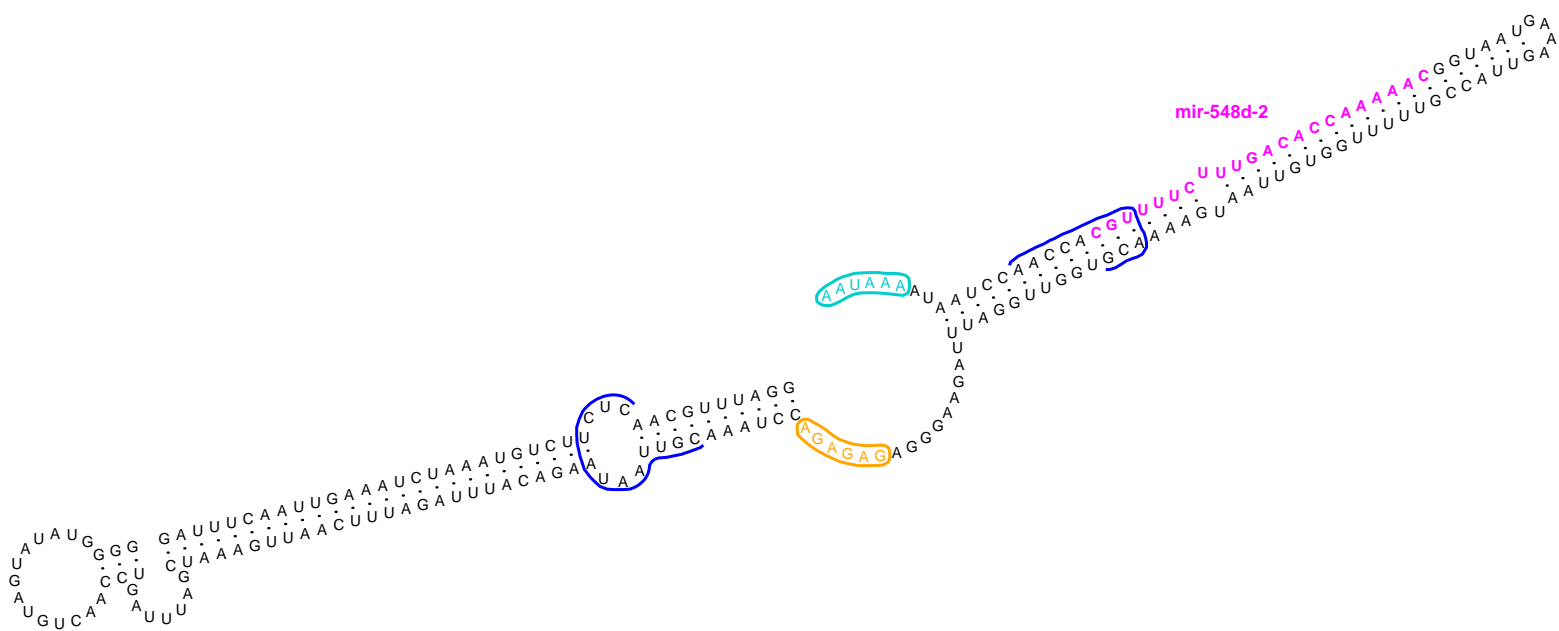

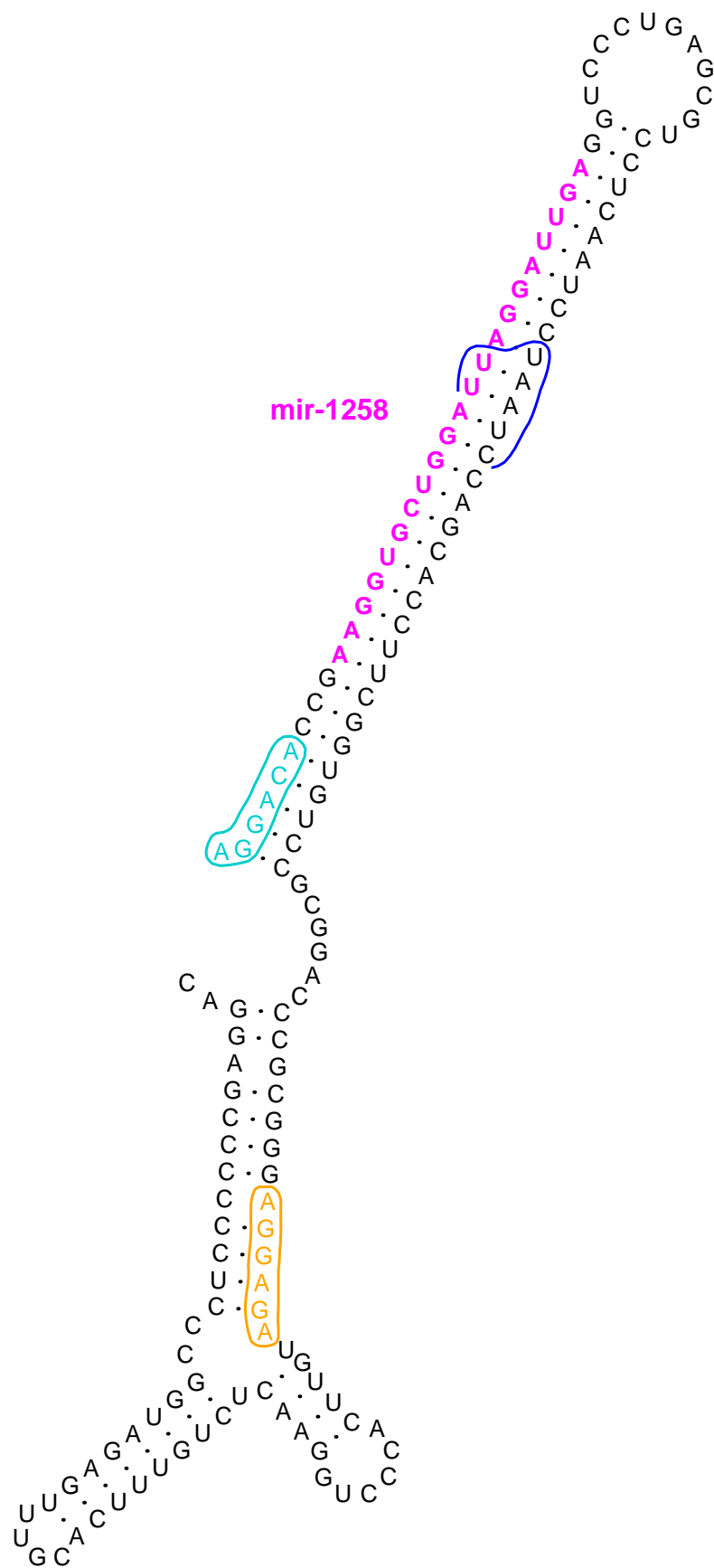

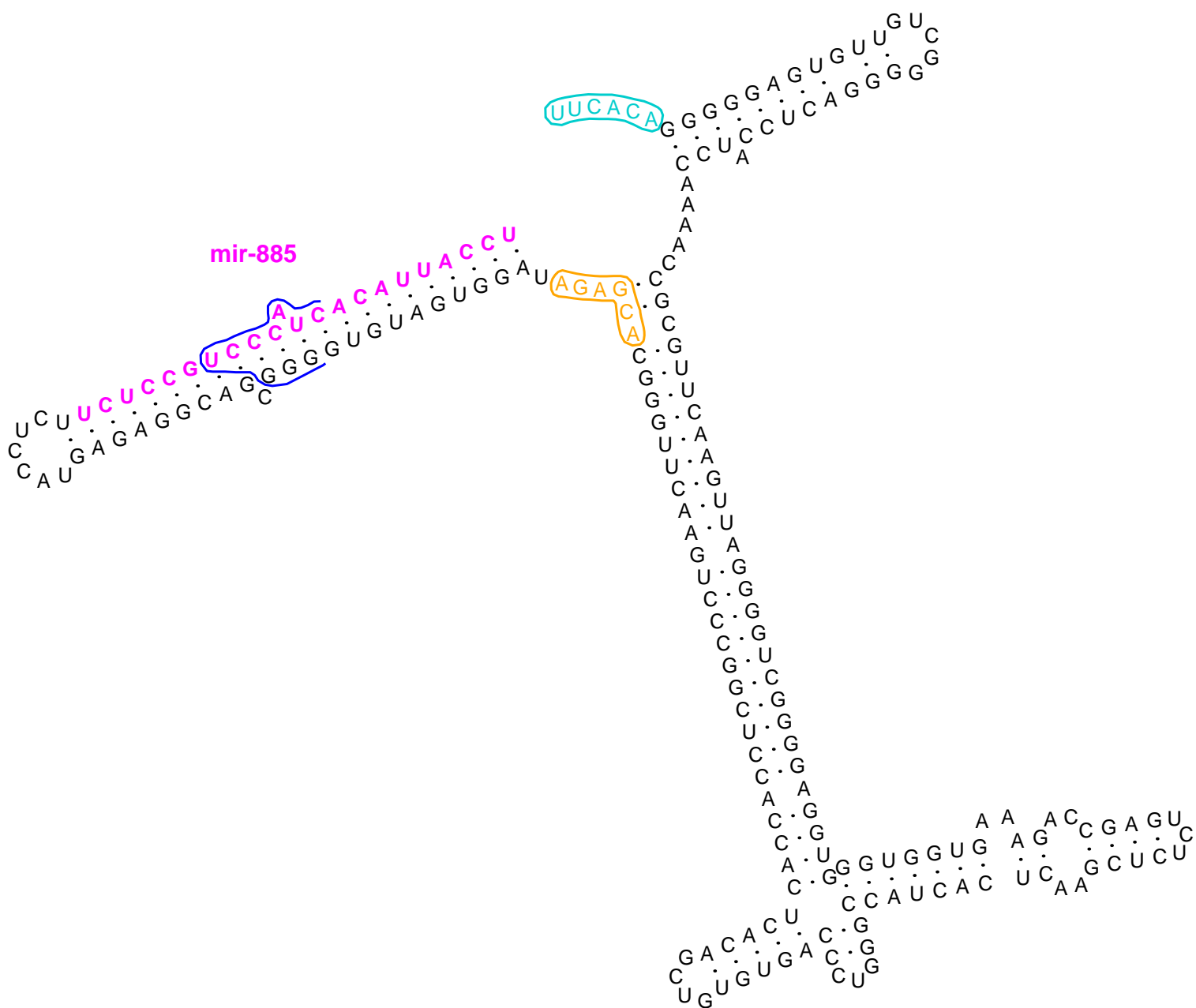

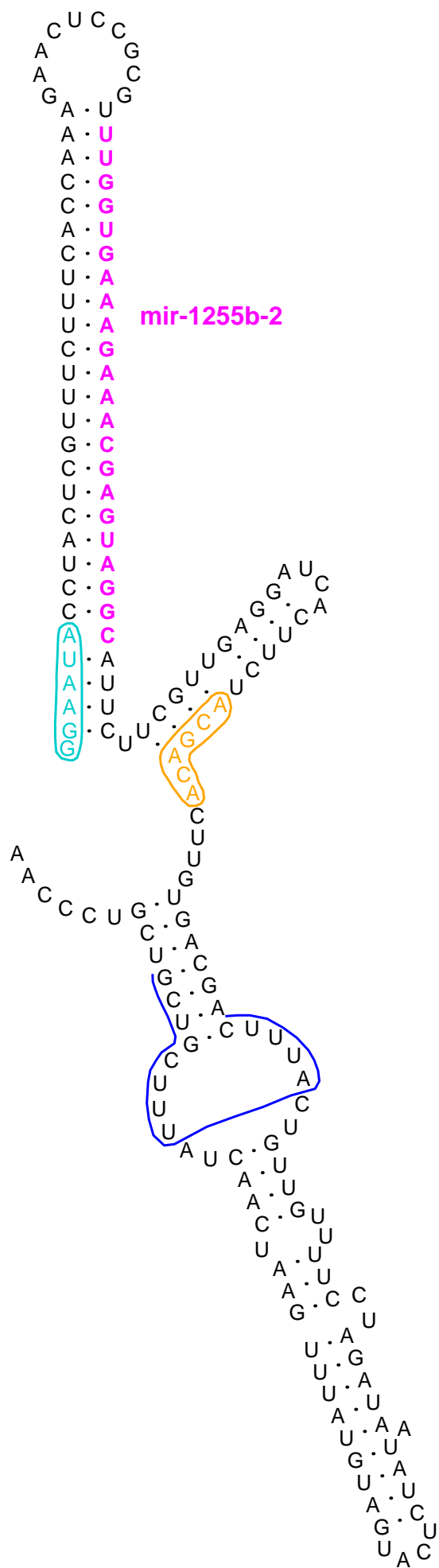

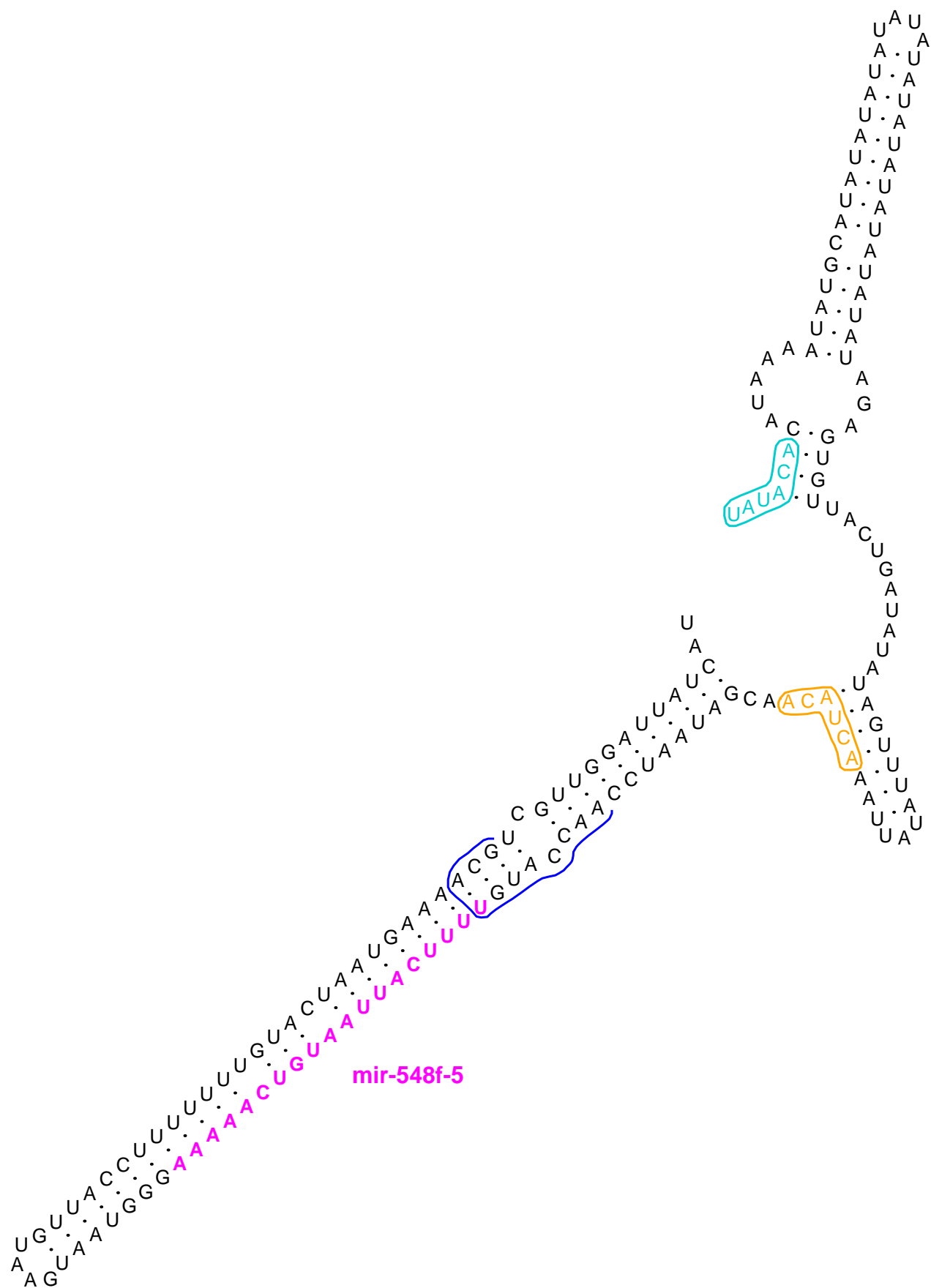

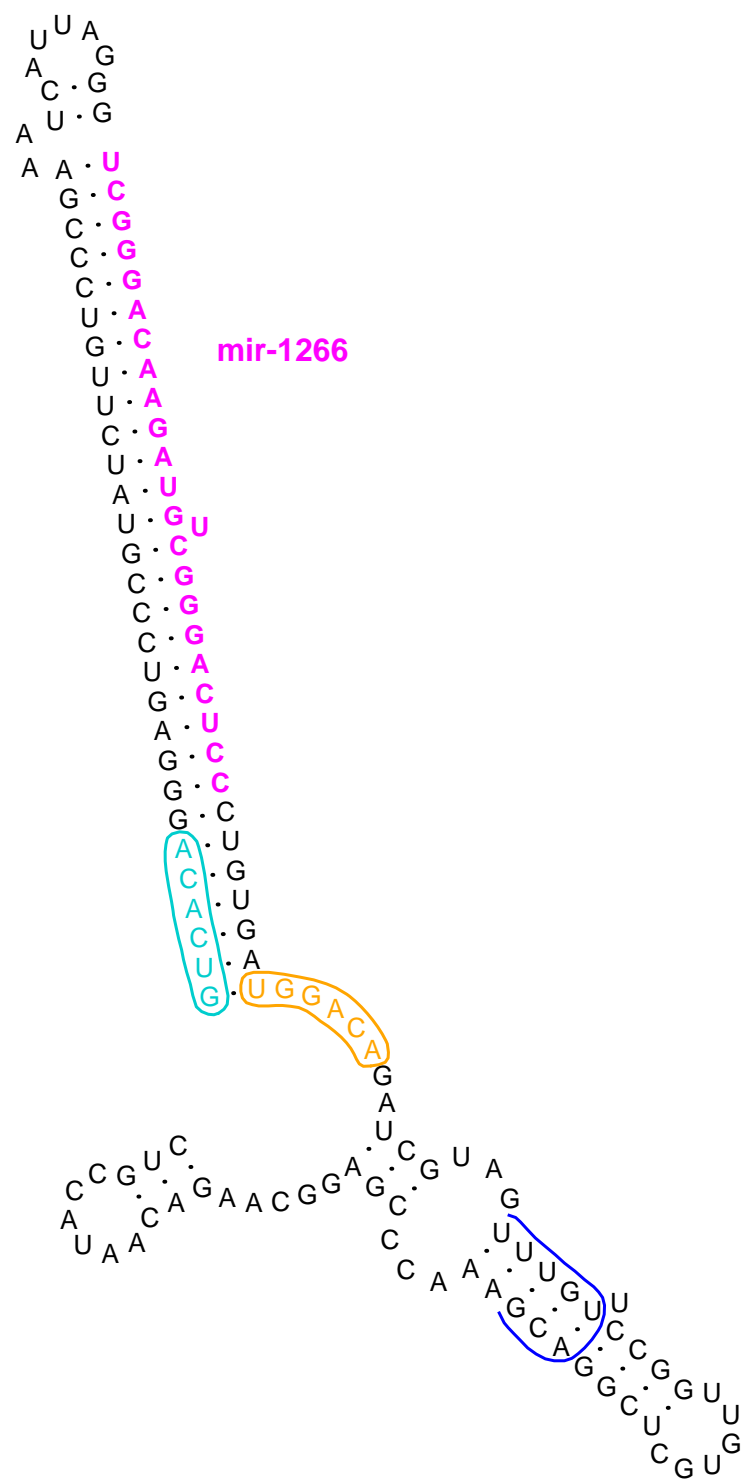

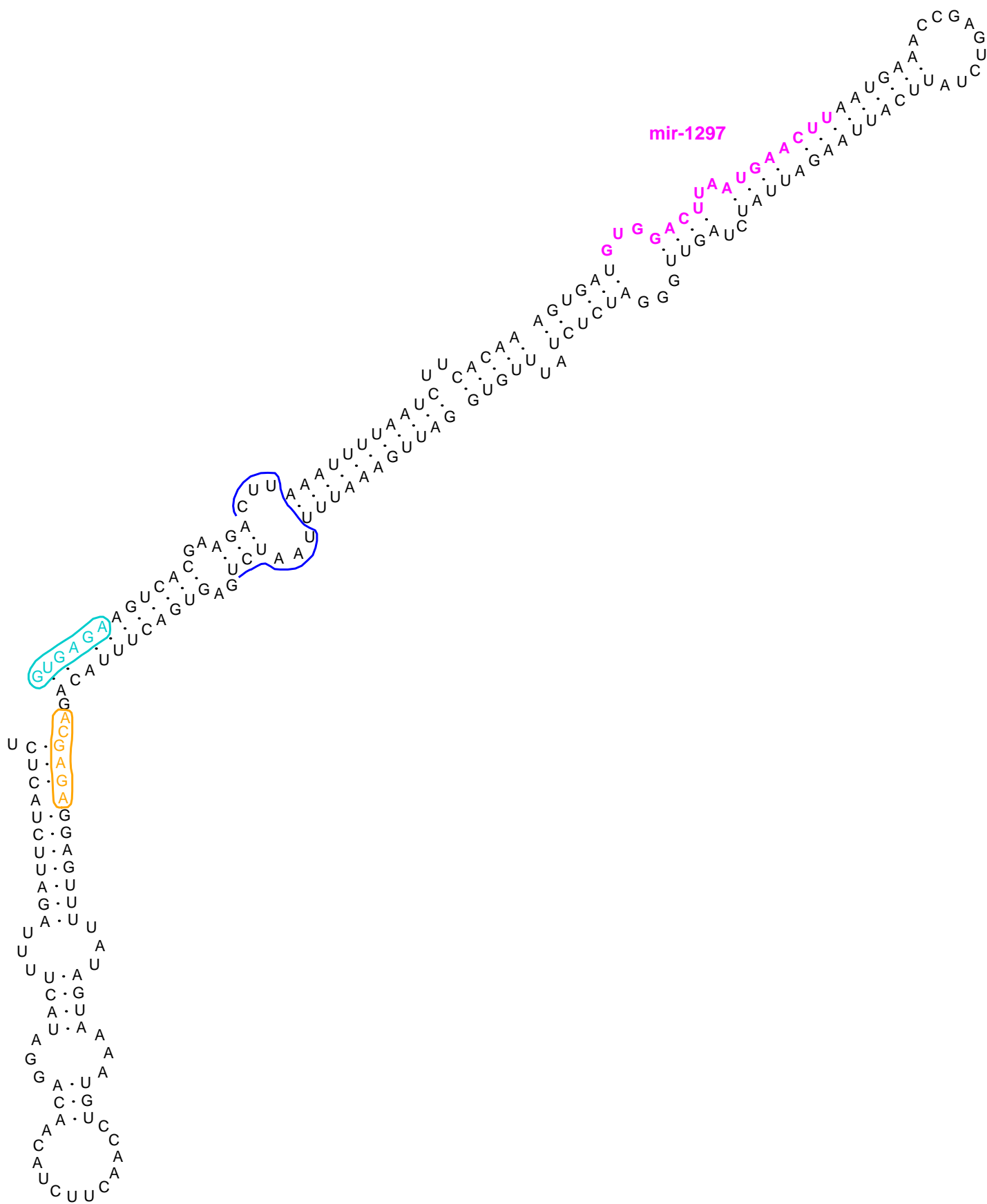

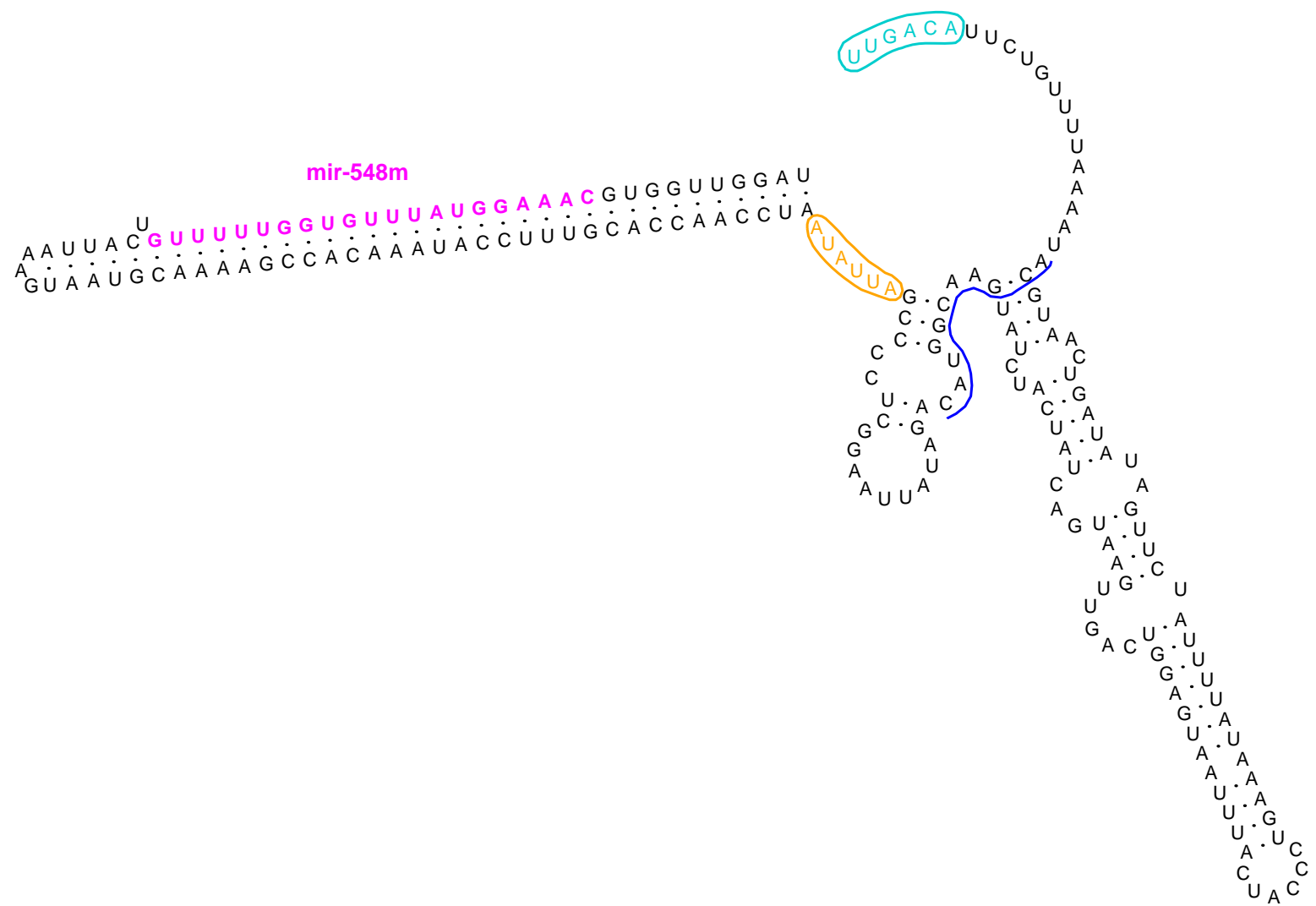

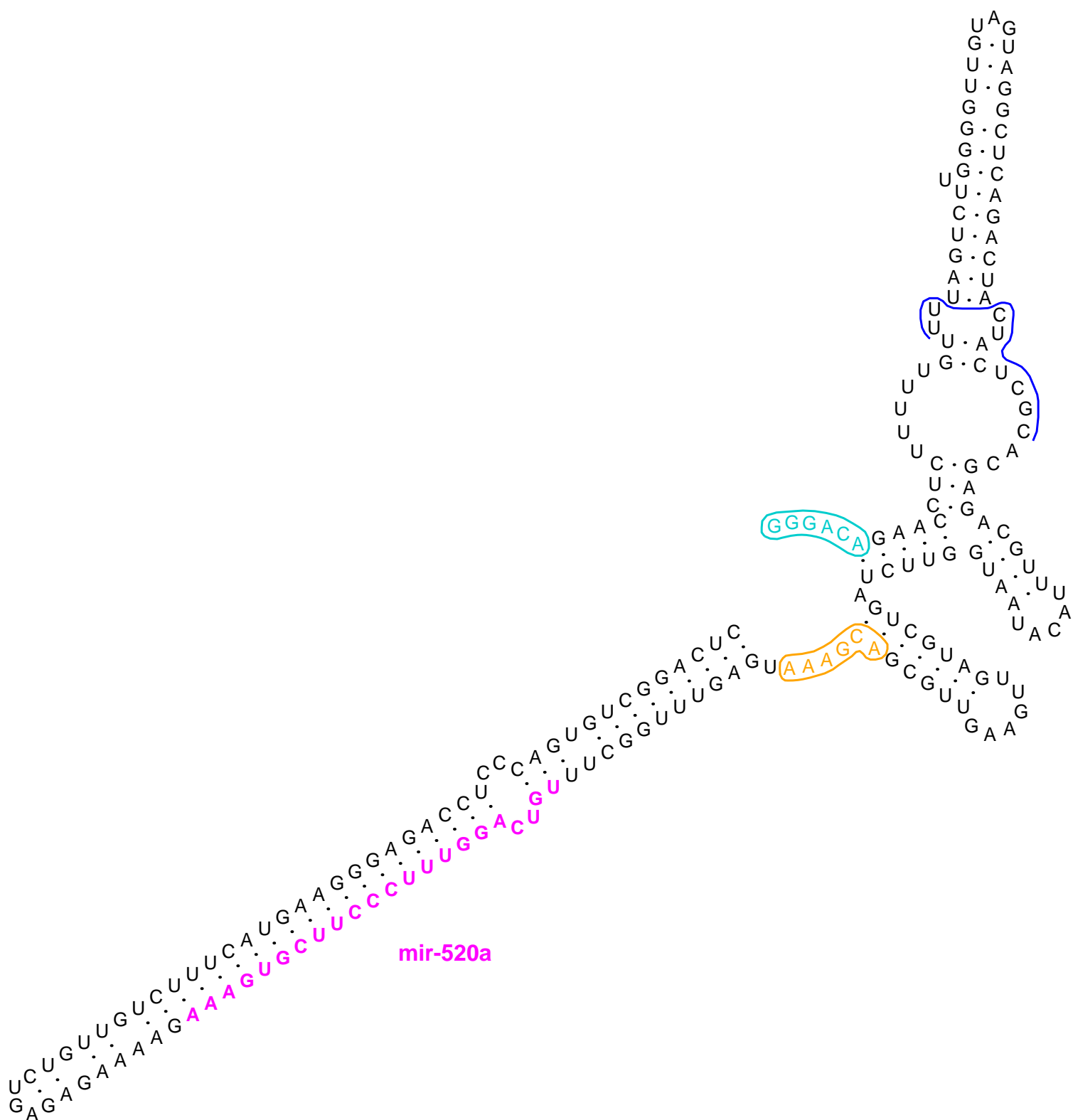

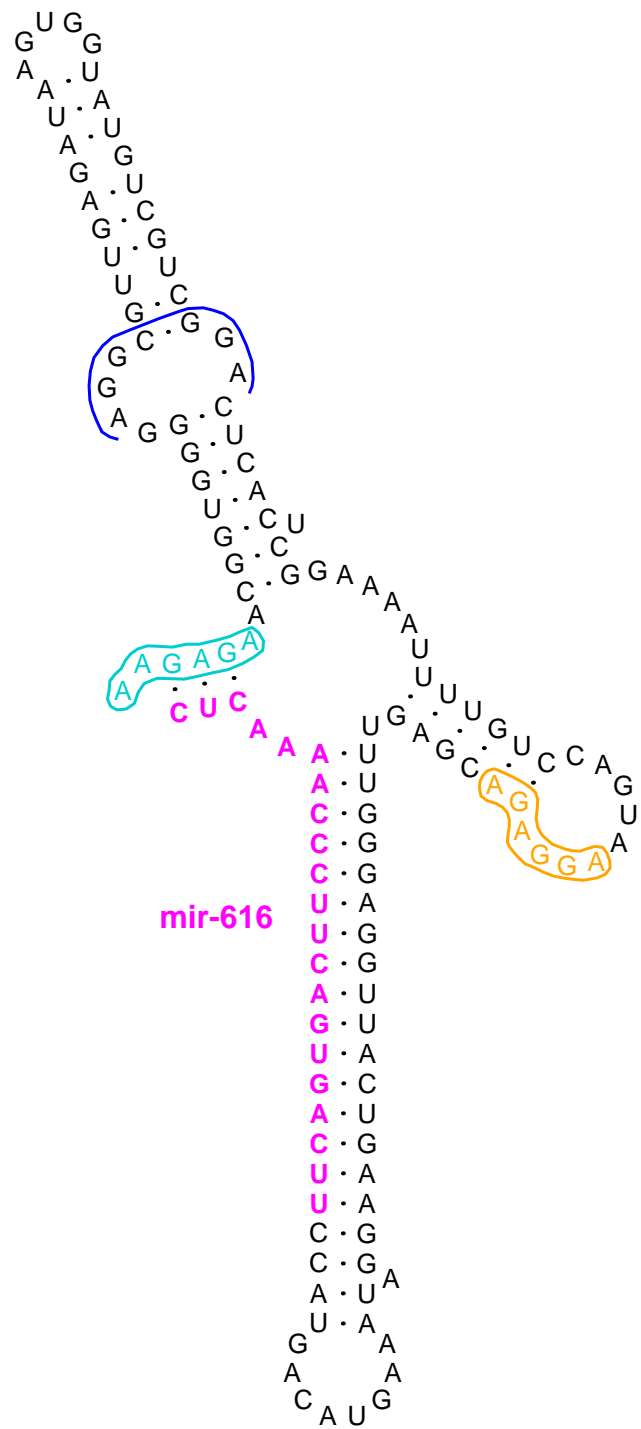

Supplement: Figure S2 — Predicted secondary structures of H/ACA snoRNA-like miRNA precursors (0.07 MB PDF) [file pcbi.1000507.s002.pdf]

A

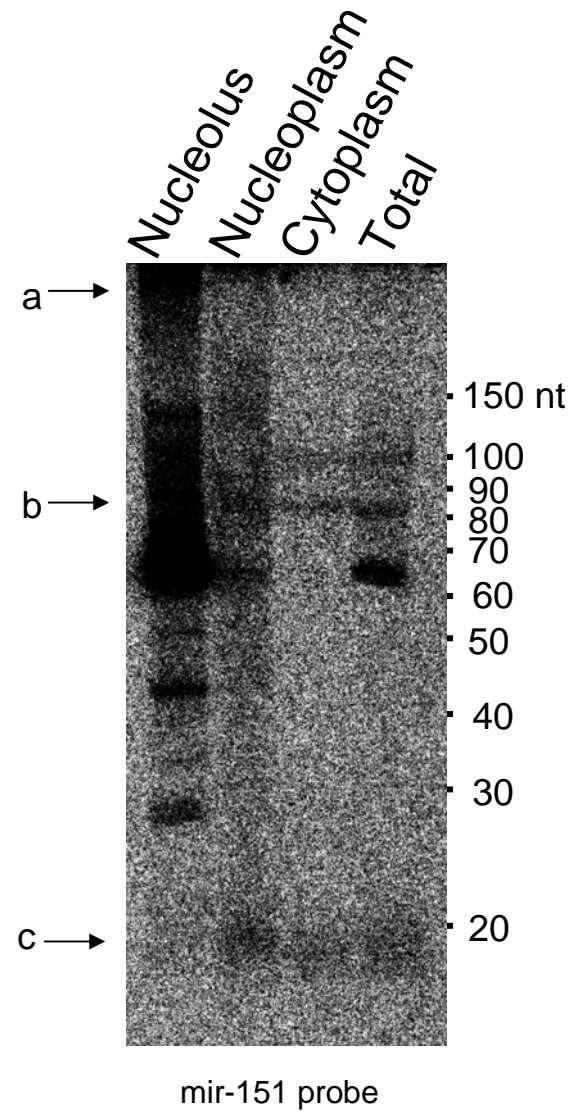

B

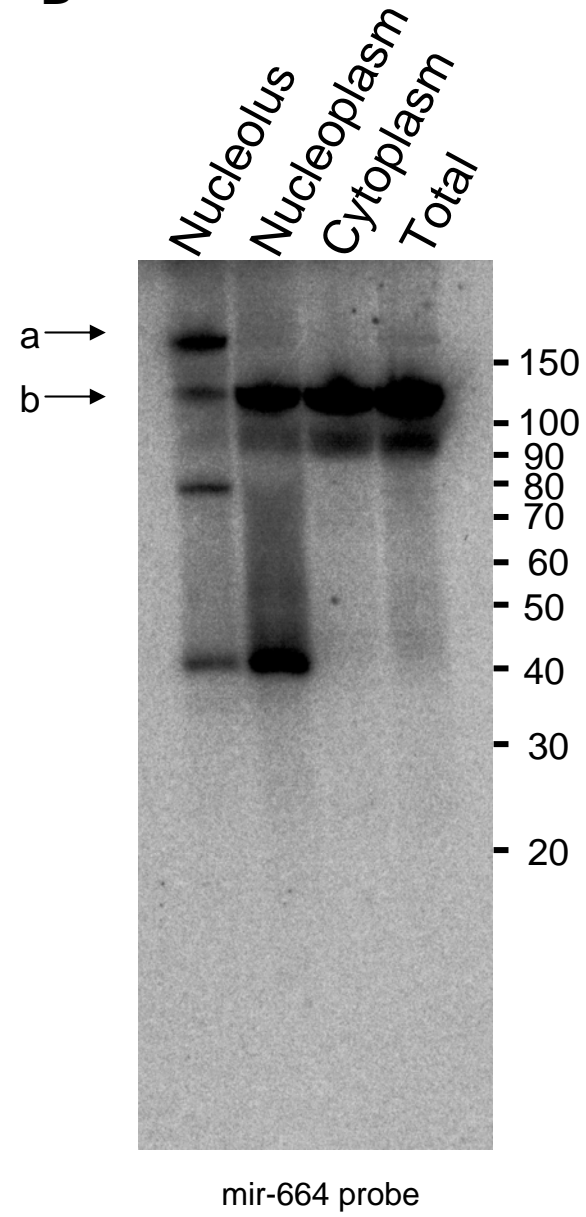

Supplement: Figure S5 — Northern blot showing the subcellular localization of H/ACA snoRNA-like miRNA precursors (same as Figure 6AB but longer exposure) (0.31 MB PDF) [file pcbi.1000507.s005.pdf]
