# Supplementary material for: Human miRNA Precursors with Box H/ACA snoRNA Features
Source: PLoS Comput Biol. 2009 Sep 18;5(9):e1000507. doi: 10.1371/journal.pcbi.1000507 (PMC2730528; doi:10.1371/journal.pcbi.1000507)
Supplement: Figure S3 — Repeat elements in proximity of H/ACA snoRNA-like miRNA genomic regions (3.58 MB PDF) [file pcbi.1000507.s003.pdf]

Screenshots of the UCSC Genome Browser displaying RefSeq genes (dark blue lines with hatch marks), miRNA hairpins (red blocks), snoRNAs (green blocks with hatch marks), repeat-elements (blue blocks with hatch marks) are shown for the genomic regions surrounding specified miRNAs.

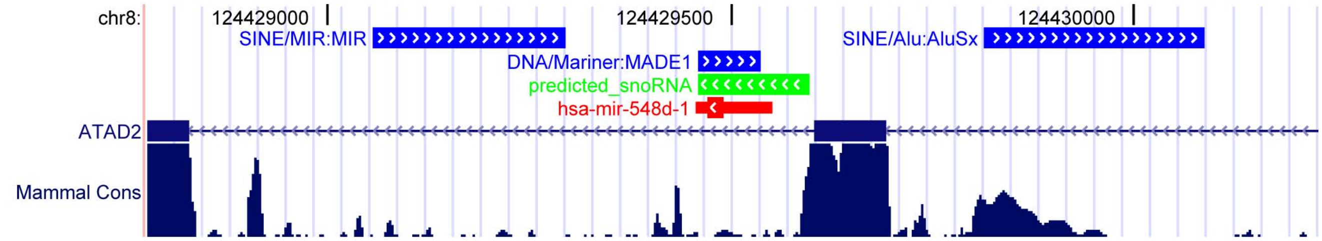

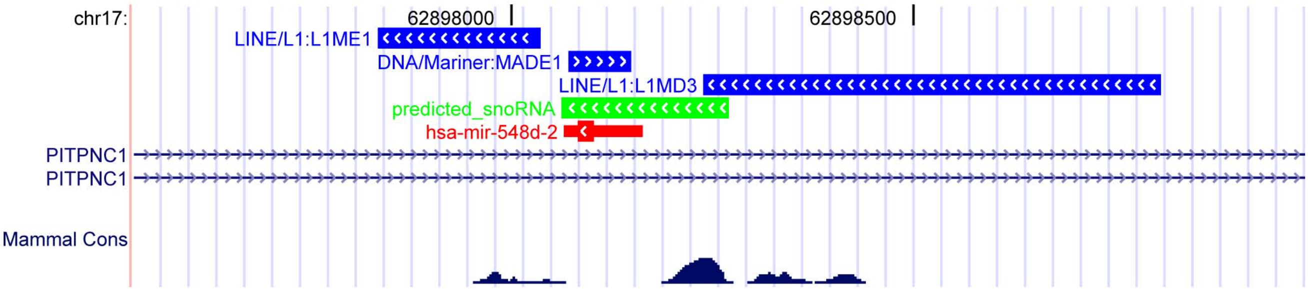

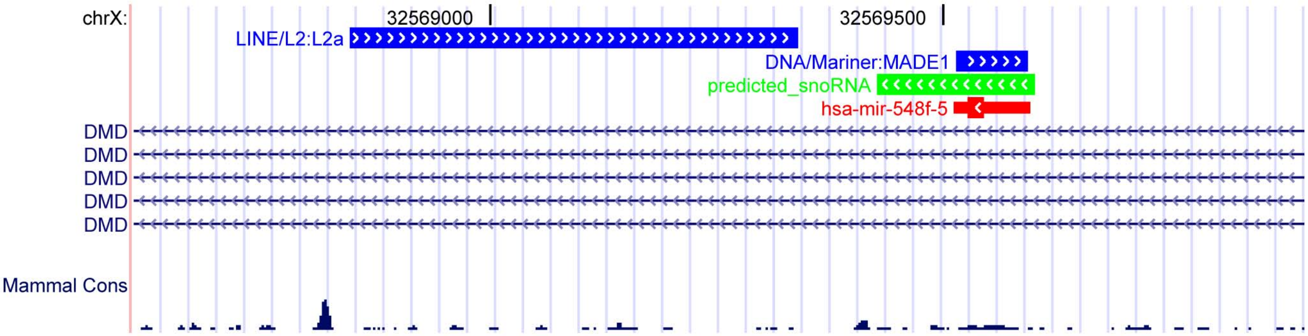

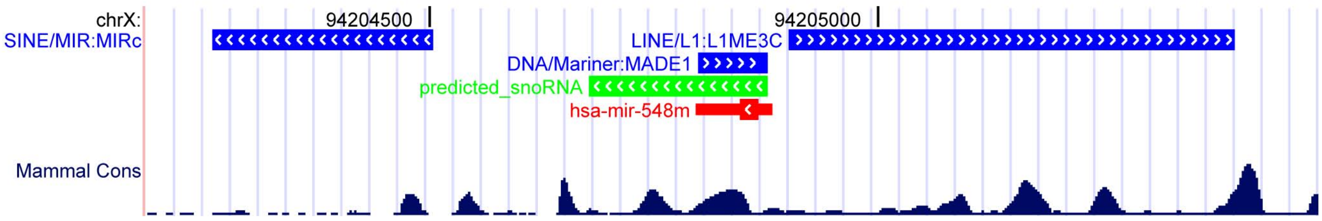

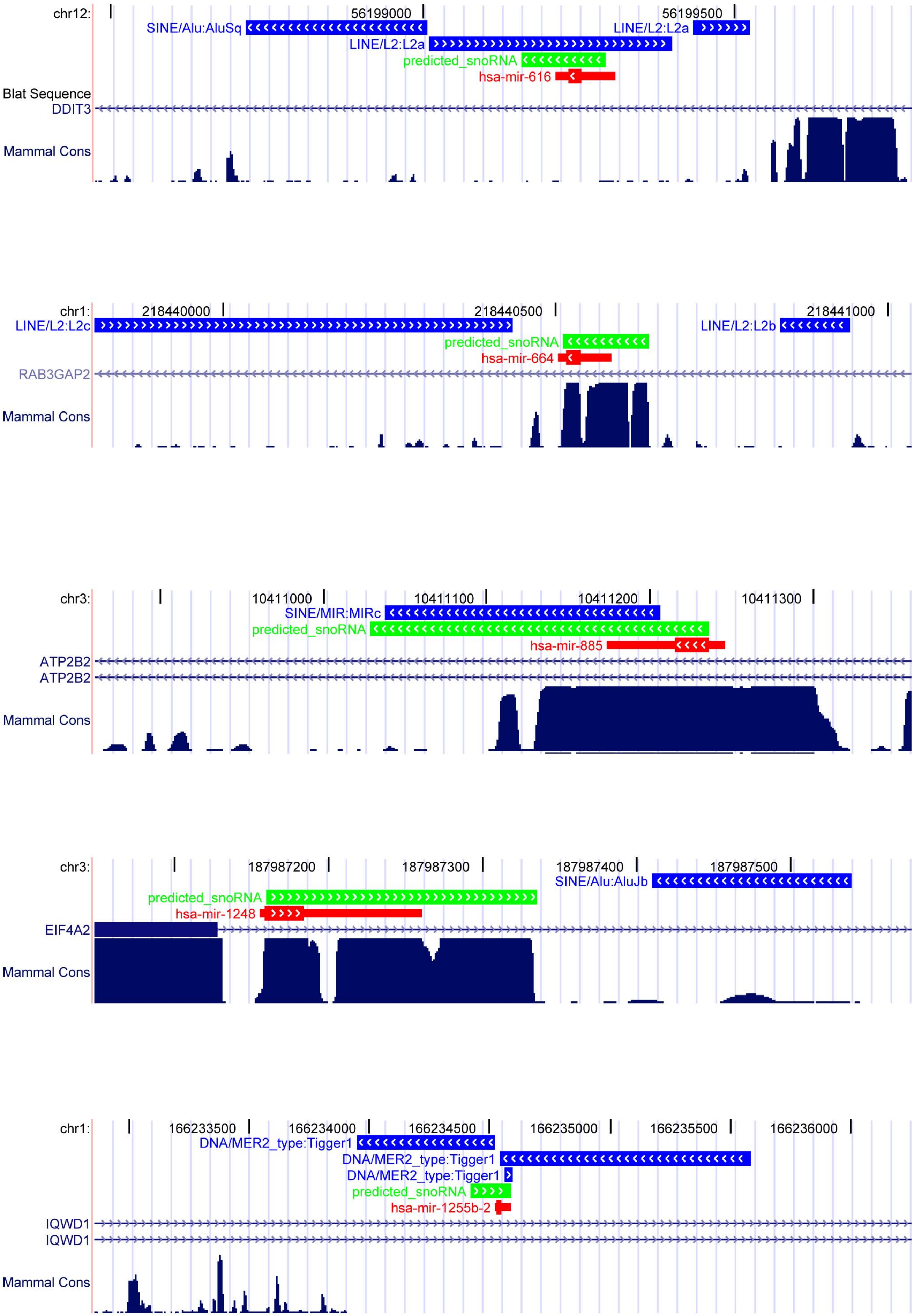
chr12:
56199000
56199500
SINE/Alu:AluSq
LINE/L2:L2a
LINE/L2:L2a
predicted_snoRNA
hsa-mir-616
Blat Sequence
DDIT3
Mammal Cons
chr1:
218440000
218440500
218441000
LINE/L2:L2c
LINE/L2:L2b
predicted_snoRNA
hsa-mir-664
RAB3GAP2
Mammal Cons
chr3:
10411000
10411100
10411200
10411300
SINE/MIR:MIRc
predicted_snoRNA
hsa-mir-885
ATP2B2
ATP2B2
Mammal Cons
chr3:
187987200
187987300
187987400
187987500
SINE/Alu:AluJb
predicted_snoRNA
hsa-mir-1248
EIF4A2
Mammal Cons
chr1:
166233500
166234000
166234500
166235000
166235500
166236000
DNA/MER2_type:Tigger1
DNA/MER2_type:Tigger1
DNA/MER2_type:Tigger1
predicted_snoRNA
hsa-mir-1255b-2
IQWD1
IQWD1
Mammal Cons

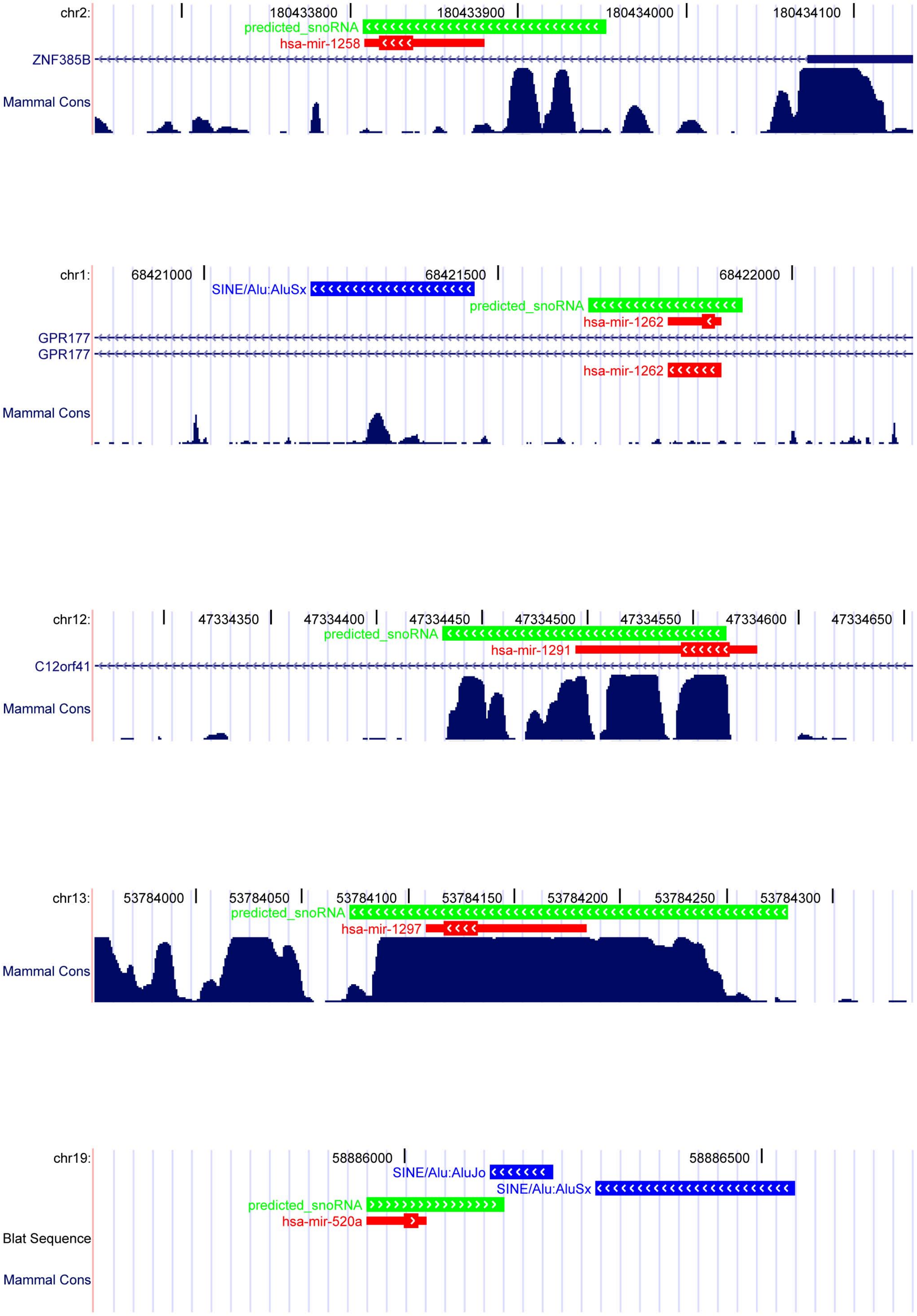
chr2:
180433800
180433900
180434000
180434100
predicted_snoRNA
hsa-mir-1258
ZNF385B
Mammal Cons
chr1:
68421000
68421500
68422000
SINE/Alu:AluSx
predicted_snoRNA
hsa-mir-1262
GPR177
GPR177
hsa-mir-1262
Mammal Cons
chr12:
47334350
47334400
47334450
47334500
47334550
47334600
47334650
predicted_snoRNA
hsa-mir-1291
C12orf41
Mammal Cons
chr13:
53784000
53784050
53784100
53784150
53784200
53784250
53784300
predicted_snoRNA
hsa-mir-1297
Mammal Cons
chr19:
58886000
58886500
SINE/Alu:AluJo
SINE/Alu:AluSx
predicted_snoRNA
hsa-mir-520a
Blat Sequence
Mammal Cons

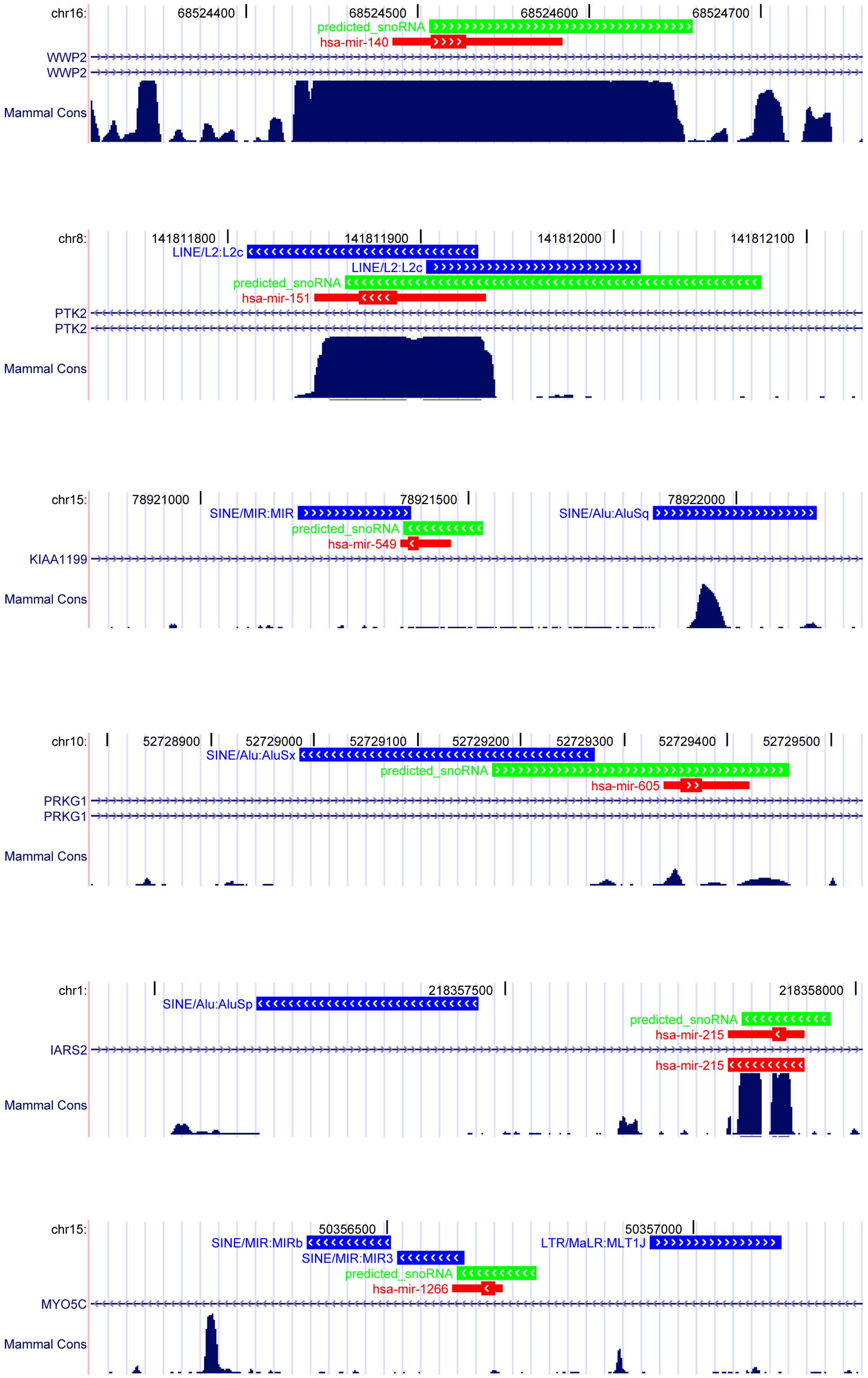

chr16:
68524400
68524500
68524600
68524700
predicted_snoRNA
hsa-mir-140
WWP2
WWP2
Mammal Cons
chr8:
141811800
141811900
141812000
141812100
LINE/L2:L2c
LINE/L2:L2c
predicted_snoRNA
hsa-mir-151
PTK2
PTK2
Mammal Cons
chr15:
78921000
78921500
78922000
SINE/MIR:MIR
SINE/Alu:AluSq
predicted_snoRNA
hsa-mir-549
KIAA1199
Mammal Cons
chr10:
52728900
52729000
52729100
52729200
52729300
52729400
52729500
SINE/Alu:AluSx
predicted_snoRNA
hsa-mir-605
PRKG1
PRKG1
Mammal Cons
chr1:
218357500
218358000
SINE/Alu:AluSp
predicted_snoRNA
hsa-mir-215
IARS2
hsa-mir-215
Mammal Cons
chr15:
50356500
50357000
SINE/MIR:MIRb
SINE/MIR:MIR3
LTR/MaLR:MLT1J
predicted_snoRNA
hsa-mir-1266
MYO5C
Mammal Cons
