# Supplementary material for: Human miRNA Precursors with Box H/ACA snoRNA Features
Source: PLoS Comput Biol. 2009 Sep 18;5(9):e1000507. doi: 10.1371/journal.pcbi.1000507 (PMC2730528; doi:10.1371/journal.pcbi.1000507)
Supplement: Figure S4 — Sequence elements surrounding selected miRNAs (0.03 MB PDF) [file pcbi.1000507.s004.pdf]

Sequence elements surrounding selected miRNAs: target site duplications (TSDs), poly(A) tails and snoRNAs.

Shown below are the genomic regions surrounding 20 of the miRNAs predicted to be encoded in H/ACA snoRNAs. Apart from the sequence of miRNA hairpin, features considered include target site duplications (TSDs), poly(A) tails, L1 consensus recognition sites and the predicted snoRNA.

Legend:
bold: TSDs
double underline: L1 consensus recognition site
wavy underline: poly(A)
single underlined: miRNA
uppercase italics: predicted snoRNA
boxed: ACA box
shaded box: H box
black background: second TSD

>mir-1291
tg**aaattgttctcattg_tag**aaggaccagtaaagtaatgtgatttgagttctgtccgtgagccttgggtagaattccagt*GGCCCTG*
ACTGAAGACCAGCAGTTGTACTGTGGCTGTTGGTTTCAAGCAGAGGCCTAAAGGACTGTCTT
CCTGTGGTCTGTTGGCTGTTCTGGGACCTCAGTAGGGAATGGCTATTTCATTTGGAAGAAAC
*AACC*tacagctcaccatcccattggaagaggctcaggaataggctcttccaagggactaggatagctaatatgggaagagcatctgt
ccagaattaatttctgtt**gtattgttcttattgctag**

>mir-664
at**aaaaatgctgcaaaa**t*TCCAAAGTGTTAAGTTCAGTTCAGGGTAGCTTCCCTGCTCTGTTAATTAA*
ACTTTGGAACATTGAAACTGGCTAGGGAAAATGATTGGATAGAAACTATTATTCTATTCATTT
*ATCCCCAGCCTACAAAA*tg**aaaaaaggtacaata**

>mir-548d-1
*A****CATTGTTTTAAGCAGTTAAAATA****AATACTACATTTATTTCAAATATGAAACAAGTTATATTAG*
GTTGGTGCAAAAGTAATTGTGGTTTTTGCCTGTAAAAGTAATGGCAAAAACCACAGTTTCTTT
*TGCACCAGACTA*ataaagaaatacaaggttatatttgtttcttatctcttatttgtggcattttcttctcatgaagtcttggtattaaataga
gatatttgacaaattggccatcaggggcagagtagataataatataattgtacaaaatacaatatattattatatcataataagataatagct
attctttactgaccattttgtagt**catccttttaagcattttacata**

>mir-151
tc**caaa*GCTCTTTGTTCTT***CTTGTCAGGCTTACCCTATGCTGCTATAGATGGCTGCAACCCGTG*
TTCAATTCAGTTCGATAAACACTGTTGAGCACCTACAGTAGCTGAGCCTGGTGCTAGTCACT
GGGGCAAAGATGACTAAAACACTTTTCCTGCCCTCGAGGAGCTCACAGTCTAGTATGTCTCA
*TCCCCTACTAGACTGAAGCTCCTTGAGGACAGGG*atggtcatactcacctcggtgttgcccaaacctccaggcc
agtagcctagctagctagatacaggaagagtcacccaggcactgaattcaaggcttagaacatatgagtgattcttaatactaaggaat
gctccactgaggtaaattatctccaaaaagtatccaatacatattttagaactacatataattactttaaataggtacctgtagtataatttatg
gatatcttcttatatactactagcacaggtagttgagcattcattcattctgcattgttctgttttagaaatggtgtttaactttgcgttttgaacatat
acaattaggctaccaaataggccaccattttatattataaaatgtcactatataatataattgcttacttggacatttgcatataacagtgtatc
acaataactaaataggttattaatttttttcaaaatttttattatttccaagattttaacaaagaaataataagtttg**gatagccctttggtcgt**

>mir-548d-2
at**aaatgtatca**taattagtacgatattaacattgggggaaggtggatgaagactacgggggttctct*GGATTTGCAACTCTT*
CTGTAAATCTAAAGTTAACTTTAGGGGGTATATGATGTCAACCTGATTTAGCTAAAGTTAACTT
TAGATTTACAGAATAATTGCAAATCCAGAGAGAGGGAAGATTTAGGTTGGTGCAAAAGTAATT
GTGGTTTTTGCCATTGAAAGTAATGGCAAAAACCACAGTTTCTTTTGCACCAACCTAATAAAA
*TAA*aaagtttaaaaggaaaa**aaatctctca**

>mir-215

at**aaaattaacaaatgatt**aagaattaatatcaattttcttaaattcaagttttgta*ACACCAAAAAGATCCAATAATGGAA*
GAGGATTAAAGTCATCATTCAGAAATGGTATACAGGAAAATGACCTATGAATTGACAGACAAT
*ATAGCTGAGTTTGTCTGTCATTTCTTTAGGCCAATATTC*tgtatgactgtgctacttcaatatcagaaatcgacta
acaccacgcaaccaacgcaatggcaggtacacagaagataatctgtaacactaccatgtaaatgtattgagtaaataaaacataaata
tacctaatgaaaacttgtgtttttctgacaaagctcatatttatttaacctaacttactgaccagtcaacctatgtaccatttaattatcaggatgt
atcaattaaaccagtttgctatatttactaatacatacatatgatccacctcagagtccagagggctattccaccaaggaatccccaccca
cctcaatcacttcagtgaaattccctagacatcttgctctctaaatagtgaaaagaagagtagccgggcacagtggctcacgcctgtaatc
ccagcactttgggaggctgaggcgggcggatcacttgaggtcgggagtttgagaccagcctgaccaacatggagaaaccccgtctcta
ctaaaaaatatatatatataaaattagccgggcgtggtggcacatgcctctaatcccaactactcaggaggctgaggcaggagaatcac
ttgaacctgggaggcggagattgcagtgagctgagatcgtgccactacactccagcctaggaaacaagagtgaaactccatctcaaa
aaaaaaaaaaaaaaaaaaaaagaagaggagacaatgacatggcttataaaactttccaaaaaaaaaaaaaactttccagtgtgaa
attttaaacaaatgttt

>mir-549
tgttt*CTATT*
CCAAGAGTGTGTTTCTAAGTTGACTGTTGAGAACCTGGCATCACATCTTCTGATA
GACATGCAACTCAAGAATATATTGAGAGCTCATCCATAGTTGTCACTGTCTCAAATCAGTGAC
*AACTATGGATGAGCTCTTAATATAT*cccaggcactgtgctaggcattttccatgcatctttgtatgtaatccccgccaggaa
ttagtatcaccatttacagatgaggaacagagttgcaggaaatttgtgcagctgtctcaaggtcttttggccagttagggaaggcgttgaat
ctaggtctgtgtgatcccaaatctcatgctttccccatatgcaa**tgttgctatt**

>mir-1258
cctctgcttgcccggcccccgccctcctccgcagagcgagcctgcacctgggcgcgccgcccgagggaatggc*CAGGAGCC*
CCCCTCCCGGTAGAGTTTGCACTTTGTCTCAAGGTCCCACTTGTAGAGGAGGGCGCCCAGG
CGCCTGTGGCTTCCACGACCTAATCCTAACTCCTGCGAGTCCCTGGAGTTAGGATTAGGTC
*GTGGAAGCCACAGGA*gcatact**cctctgttccccagg**

>mir-885
gg**aaatgcaggact**agactagatgccatcaatgtggcagtggaagggcgtggagccagggggccaggatggcggggctgtcttca
aaaaacagcaaggcctggctccagcaggcccgatggctcaaccccgcgagaccccgacacaccctcctcccttccccctgccagc
cagagcactaacagcatgaggtcatgcaggacatcggtaaaccctcttgggactctgcccacatcccaggacctgctgcccacccac
caagcagaaggccaggcatgggagagccggacctgccaccactgccaccgtctccagggagcctaggacgacaccgtgactgca
gaaggcccagtctgctactcggcccgcactctc*TCCATTACACTACCCTGCCTCTTCTCCATGAGAGGCAGC*
GGGGTGTAGTGGATAGAGCACGGGTTCAAGTCCCGGCTCCACCACTCACAGCTGTGTGAC
CCTGGGCCATCACTCAAGCTCTCTGAGCCAGAAAGTGGTGGGTGGAGGGGCTGGGGATTG
*AACTTGCGCCAAAACCTACCTCAGGGGGCTGTTGTGAGGGGGACACTT*tgcctactgcctcgtgctctg
tgagcgtgtgttattaactgcattaataacaataat**aatggaaggact**

>mir-140
ct**aaaaagagtccaaa**gcattgtcttgccttcaccctggtccacccataaaagttaacaggccctctgggaaatgctgccttggacagc
agggcagaagggccccggcagagagagagagcgctgtggcccgcagcgacctctccgctcgagacctctcccggtgtgggcatgtc
ttcccgcttggtgttgggttaacttgcttctggtcctctgttcggtggtggcgttgccttctgcgctggccccgtggatggatgttcctttttccgtg
gtgacctcctctccaggctctgcttggtgggcttctggtgtggccgcgccctgtgtgtgtctctctctgtgtcctgc*CAGTGGTTTTACC*
CTATGGTAGGTTACGTCATGCTGTTCTACCACAGGGTAGAACCACGGACAGGATACCGGGG
CACCCTCTGCGTCGACGGACTCCTCGTCTGCCCAGCCACAAACAGCCCAGCAGCTGGGGA
*CTGGCCCCCTTGGGACATCC*ccttgctggtttagctcaagccc**aaaaggactctgaa**

>mir-1248
tt**acaatacaa___ctgatgtgttttgttgtcgttccccctgcttaaa**gcacttgatgcataactctgtctaccttcattccgtagtaagac
agagacgcttggcttcagacattttcctttgggtattaatgtgtaagttgtgctacaacataattttctctttttaaggttgtgttgctttctgccaca
atgccaactgatgtgttggaagtgaccaaaaaaattcatgagagatccaattcgaattctggtgaaaaaggaagaattgacccttgaagg
aatcaaacagttttatattaatgttgagagagaggtaactgtctgattgttagacattatttta c*CTTCTTGTATAAGCACTGTGC*
TAAAATTGCAGACACTAGGACCATGTCTTGGTTTTTGCAATAATGCTAGCAGAGTACACACAA
GAAGAAAAGTAACAGCACTAGATTGTAAAGACTGGGGTGGACCTCTTTCTTAATGTCCAATG
*TCCTTTGTCTTAAGATTTGGTGCAATATCT*cattagacctaacagaatgaagttaatttttataatttctacgttttgagac
tgggttatgagactggctaagactggccaattttggtattttgggtagagacagtttcactttcttgaaaccctggtctggttggaactctgggc

tctaagtgctaggatcccaaagggctgggattacaggcattagcactgtggctggcccagaatgaatttttaactgaagagttggaaaaa
ctaaatgactgttaactttaacttctaaacattacaggaatggaagttggatacactttgtgacttgtacgagacactgaccattacacaggc
tgttatttttctcaatacgaggcgcaaggtggactggctgactgagaagatgcatgccagagacttcacagtttctgctctggtaagaggtg
ttctaaaatgtctggatttccactaaagcaggattcagact**acaatatagctgct_aagtgctgtgttgtcgttccccctgcttaaa**

>mir-1255b-2
cc**aaattggagtcagtcctttcaa***ACCCTGCTGCTGCTTTATCAACTAAGTTTATGTAGTACTCTATAAT*
AGATCCTTTTGTTGTCATTTCAGCAGTGTTCACAGCATCTTCACTAGGAGTTGCTTCTTACGG
*ATGAGCAAAGAAAGTGGTTTGCGCCTCAAGAAACCACTTTCTTTGCTCATCCATAAGG*agcaac
tccttatctgttagttttatcatgagattgcaacaattctcacaccttcagtctgtacttctaattctagttcttttgttgttttcacctcatctgcagtga
cttcctctactgaagtcttctacccctcaaagtgatccactagggttggaattaacttcttccaaactcctgttaatattgttattttgacctcccac
gaatcacaaaagttcttaataaggcatct**agaatgg--tcaatcctttcta**

>mir-548f-5
tt**agcaattattaat**cctatctaccaacatact*TACTATTAGGTTGCTGCAAAAGTAATCATGTTTTTTTCCATT*
GTAAGTAATGGGAAAAACTGTAATTACTTTTGTACCAACCTAATAGCAACATCAAATTATATTT
GATATATAGTCATTGTGAGATATATATATATATATATATATATATATACGTATAAAATACACATA
*T*atctctctatatg**aacaaatattaat**ggaat

>mir-1262
//start of predicted sno:
*GAATTTCATCCTTCT**ACAAATTCACCCCT**CACATTATCTACAATGGTGATGGGTGAATTTGTA*
GAAGGATGAAAGTCAAAGAATCCTTCTGGGAACTAATTTTTGGCCTTCAACAAGAATTGTGAT
ATTTAATTCTGTGTTTTCTGATGCTTCTGAGGAAAGCTCCATAAAGAGAGCTGAATGGGGCA
GAGGATTTTATACACTATAAGAACTATCCCTGCTATGATGGAACAGGCCATTGGTAGTATGAT
*ATTCACATAATT*gtatttctgtgcttcagcagatagtccttattaataaaatatgttgaagcaactgctaagaagaaatgtataactct
actctcccacccctactactactcccaccaaaaagaggtcttctttcatttacgaccatgtcttctttaatttatgactatgttatagcctgtgttaa
aactatctaacattttggctgggcacagtggttcacacctgtaatcccagcacattgggaggctgaggtgggtggatcacctgaggtcag
gagttcaagaccagcctgaccaatatggtgaaaccctgtctatacaaaaattagcccagcatggtggcgtgtgcctgtaatcccagctac
ttgggaggctgaggcaggagaatcacttgaacctgggaggtggaggttgcagcaagccgagatcgcaccactgcactccagcctgg
gcaacagagcaactctgtctcaaacaaaaaaaacctctaacattctaattagatagttttcattgttggctagtgtcactagaattttaaagtt
accttttaagtttctttaccacaatttgagtcaatgcaagcagaaataaaaact**tcaaattctccctt**

>mir-1266
ct**aaagagccagtt**tctccttggaggtctgtctgtgtaa*CTGCCATAACAGAACGGAGCCCAAAGCAGGCTCGT*
GTTGGCCTTGTTTGATGCTAGACAGGTAGTGTCCCTCAGGGCTGTAGAACAGGGCTGGGAT
*TACTAAAGCCCTGTTCTATGCCCTGAGGGACACTG*agcatgtcatttcacttctcagagtcttcatcttctctctttga
aatgaaaatattggaccggggctctgtatccaaagtctcctctagcatttacatgggaggctgtggctctgtgctctgggtcaagtcactcc
acccctgagtcaccattttactcttctgtaaaagagatatctatgtcgtaggatttttgtgaagcttaaatgagaatctgtattcacccagcacc
tggcatgtgagcggtgccacatcc**acagagccatgtt**

>mir-1297
tt**tatatgttgca**tgtaatgtatcatacataatgtgtt*TCTCATCTTAGATTTTCATAGGACAACATCTTCAACCTGT*
AAAATGATATTTTGAGGAGAGCAGACATTTCAGTGAGTCTAATTTTAAAGTTAGGTGTTTATC
TCTAGGGTTGATCTATTAGAATTACTTATCTGAGCCAAAGTAATTCAAGTAATTCAGGTGTAG
*TGAAACACTTCTAATTTTAAATTCAGAAGCACTGAAGAGTG*agttggattattggctgtagcacttacttgatcct
ctttcttatcacccttgacttttaaagcctggctttaaaaaaa**tatatgcttca**

>mir-548m
tt**atatttcttaaatttacgattt**tattttcttgttggttcatatgagtttttttgttggtgtttattctagatat*TAGGTTGGTGCAAAGGT*
ATTTGTGGTTTTTGTCATTAAAGTAATGCAAAAGCCACAAATACCTTTGCACCAACCTAATATT
AGCCCCTCGGAATTATAGACATGGCAAGTATCTACTATCAGTAAGTTGACTGGAGTAATTTAC
*TACCCTGAAATATTTTATCTTGATATAGTCAATGCATAAA**ATTTTGTCTTACAGTT***tgtgattt**

>mir-605

acctgtctttttgcctttgatgaatttattgcttaagactttagccagtatctctttttgcccattcttttatctgtatttattttatatgtttttgagatggagtctcactgtgttacccaggctagagtgcagtggtacgatctcggctcactgcaacctccgcctcctgggttcaagcaattctccttcctcagcctcctgaataggtaggattacaggcatcctccaccatgcctggctaagttttgtaattttagtagagatggggtttcaccat*GTTGGCCAGGCTGGTCTTGAACTCCTGATCTCAGGTGATCCACCCACCTCGGCCTCCCAGAGTGCTGAGATTGCAGGTGTGAGCCACCATGCCCCGCCTCTTTTTGCTCATTCTTTAAAGTAGGAACTTTGGTAGAACTTTCACAGCCTGTAACATATGTCTCTAGCCCTAGCTTGGTTCTAAATCCCATGGTGCCTTCTCCTTGGGAAAAACAGAGAAGGCACTATGAGATTTAGAATCAAGTTAGGACTGCAGATACAGGTTACCTATGTTACAGGCTGACAGCC*acaggtatattgctctctgc**acatgtgttttttcct**

>mir-520a
at**aaacccagagtgctggagc**aagaagat*CTCAGGCTGTGACCCTCCAGAGGGAAGTACTTTCTGTTGTCTGAGAGAAAAGAAAGTGCTTCCCTTTGGACTGTTTCGGTTTGAGTAAAGCAGCGTTGAAGTTGATGCTGATCTTGGTAATACATTTGCAGAGCACGCTCATCATCAGACTCGGATGATGTTGGGGTTCTGATTTTGTTTTTCTCCAAGACAGGG*tctctgttgcccaggctggagtgcagtggcagttccaacctagctctcctgggcccaagagaccctctatttatttatttatttatttatttatttatttattcattcattcatttagagatggaatctggctctg**tgacccaggctggag**

>mir-616
tc**cattcattcttaacaaat**acttaatgaggacctactgtgtgccacacagtttggggctcagggtacatccttgagcaagaggaaaaaatcatctcagtgggaggcctacagtaaacaaaatataagtgccacggagaaagctaaagcagagaaaggaatggagaatgttcaggatggaggtcagagtgttacatcaggtggtcaggaattaccttaggtaattcctcca*CTCAAAACCCTTCAGTGACTTCCATGACATGAAATAGGAAGTCATTGGAGGGTTTGAGCAGAGGAATGACCTGTTTTAAAAGGCTCACTCAGGCTGCTGTATGGTGAATAGAGTTGCGGAGGGGTGGCAAGAGAA*gaaatgggaagaccttctgcagtcagaaagtttctgcagtaatttagagatggtagtgaattgatctagattggaaacaatggaattagaagtgtttagattcttctaagcaaaggttttaaa**aactcatttttaaagaat**
